# Peroxisome proliferator-activated receptor gamma coactivator-1 (PGC-1) family in physiological and pathophysiological process and diseases

**DOI:** 10.1038/s41392-024-01756-w

**Published:** 2024-03-01

**Authors:** Lu Qian, Yanli Zhu, Chao Deng, Zhenxing Liang, Junmin Chen, Ying Chen, Xue Wang, Yanqing Liu, Ye Tian, Yang Yang

**Affiliations:** 1 Xi’an Key Laboratory of Cardiovascular and Cerebrovascular Diseases, Xi’an No.3 Hospital, The Affiliated Hospital of Northwest University, Northwest University, Xi’an, 710021 China; 2 Xi’an Key Laboratory of Innovative Drug Research for Heart Failure, Faculty of Life Sciences and Medicine, Northwest University, 229 Taibai North Road, Xi’an, 710069 China; 3 Department of Cardiovascular Surgery, The First Affiliated Hospital of Xi’an Jiaotong University, 277 Yanta West Road, Xi’an, 710061 China; 4 Department of Cardiothoracic Surgery, The First Affiliated Hospital of Zhengzhou University, 1 Jianshe East, Zhengzhou, 450052 China; 5 Department of Hematology, The First Affiliated Hospital of Xi’an Jiaotong University, 277 Yanta West Road, Xi’an, 710061 China

**Keywords:** Pathogenesis, Diseases

## Abstract

Peroxisome proliferator-activated receptor gamma coactivator-1 (PGC-1) family (PGC-1s), consisting of three members encompassing PGC-1α, PGC-1β, and PGC-1-related coactivator (PRC), was discovered more than a quarter-century ago. PGC-1s are essential coordinators of many vital cellular events, including mitochondrial functions, oxidative stress, endoplasmic reticulum homeostasis, and inflammation. Accumulating evidence has shown that PGC-1s are implicated in many diseases, such as cancers, cardiac diseases and cardiovascular diseases, neurological disorders, kidney diseases, motor system diseases, and metabolic disorders. Examining the upstream modulators and co-activated partners of PGC-1s and identifying critical biological events modulated by downstream effectors of PGC-1s contribute to the presentation of the elaborate network of PGC-1s. Furthermore, discussing the correlation between PGC-1s and diseases as well as summarizing the therapy targeting PGC-1s helps make individualized and precise intervention methods. In this review, we summarize basic knowledge regarding the PGC-1s family as well as the molecular regulatory network, discuss the physio-pathological roles of PGC-1s in human diseases, review the application of PGC-1s, including the diagnostic and prognostic value of PGC-1s and several therapies in pre-clinical studies, and suggest several directions for future investigations. This review presents the immense potential of targeting PGC-1s in the treatment of diseases and hopefully facilitates the promotion of PGC-1s as new therapeutic targets.

## Introduction

Peroxisome proliferator-activated receptor (PPAR) gamma coactivator-1 (PGC-1) family (PGC-1s) consist of three members, namely PGC-1α, PGC-1β, and PGC-1-related coactivator (PRC). The first member to be discovered was PGC-1α, which plays important roles in modulating mitochondrial functions in brown adipose tissue (BAT) and skeletal muscle.^1^ The amino acid sequence of these three members shares considerable homology in both the N- and C-terminal ends of the proteins, which partially explains their similar features and functionalities. Originally, PGC-1s were acknowledged as pivotal regulators in mitochondrial function and energy metabolism. They exert significant roles in mediating oxidative phosphorylation (OXPHOS), fatty acid/lipid metabolism, and reactive oxygen species (ROS) detoxication.^2–4^ Considering their intrinsic capacity to coordinate cellular bioenergetics, it is not surprising that PGC-1s have diverse functions in a diverse array of diseases, such as but not limited to cancers, cardiovascular diseases, and neurological disorders. PGC-1s achieve these by activating coactivated genes such as estrogen-related receptors (ERRs), PPARs, and nuclear respiratory factors (NRFs).^5–9^ Importantly, with the development of research in the past two decades, increasing evidence supported the potential application of targeting PGC-1s therapies.^10–12^

In this paper, our aim is to provide a systematic and comprehensive summary of the architecture, upstream signals and parallel partners, biological function, and relation to health and diseases of PGC-1s. Furthermore, we also provide insights into the therapy targeting PGC-1s and suggest directions for future investigations. The compilation of information in this paper serves as a comprehensive repository, with the hope of illuminating the possibility of PGC-1s as novel therapeutic targets in the future.

## Introduction and function Of Pgc-1s

### The discovery history of PGC-1s

**Fig. 1:**
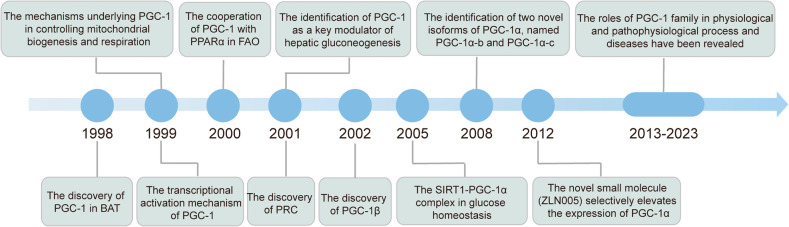
A brief history of the PGC-1s family. The figure describes the milestones of PGC-1s from the origin of different members to the most advanced scientific discoveries, including the identification of biological functions, development of activator, and recent progresses in human health and diseases

The history of PGC-1s can be traced back to its founding member, PGC-1α, which was identified in 1998 as a transcriptional coactivator of PPARγ in BAT, where it drives adaptive thermogenesis^1^ (Fig. 1). Subsequent studies revealed that the docking of PGC-1 to PPARγ stimulates a conformational change in PGC-1, which permits binding of SRC-1 and CBP/p300, thus resulting in increased transcriptional activity.^13^ In addition, Wu et al. elucidated the mechanisms by which PGC-1 controls mitochondrial biogenesis and respiration.^2^ PGC-1 was also recognized as a key modulator in fatty acid oxidation (FAO) and hepatic gluconeogenesis^14,15^ (Fig. 1). Two other members of PGC-1s family, PGC-1β and PRC, were discovered through sequence homology searches^16,17^ (Fig. 1). In 2008, the two novel isoforms of PGC-1α, PGC-1α-b and PGC-1α-c, were first identified. These isoforms are shorter than PGC-1α by 4 and 13 amino acids, respectively, and are transcribed by a novel exon located 13.7 kb upstream to the previously reported exon of the PGC-1α gene.^18^ In this text, unless the variant is specifically specified, “PGC-1α” refers to the original PGC-1α gene/protein. In 2012, Zhang et al. discovered a novel small molecule, known as ZLN005, which selectively elevates the expression of PGC-1α.^19^ However, despite extensive studies on the association between PGC-1s and various physiological and pathophysiological process and diseases, no drugs targeting PGC-1s have achieved the application from bench to bedside. Therefore, a more comprehensive understanding of PGC-1s is necessary to improve PGC-1s-related therapies for the precise intervention and management of different diseases.

### The structure of PGC-1s

PGC-1α and PGC-1β have the highest sequence identity, particularly in several domains including the N-terminal activation domain (40% homology) and the C-terminal RNA binding domain (48% homology).^20,21^ They are both predominantly expressed in tissues that demand high energy consumption, such as BAT, heart, and brain.^1,16^ PRC is expressed in all tissues and shares lower levels of homology compared to the other two members.^17^ It remains poorly characterized and known, greatly because of the embryonic lethal phenotype of PRC knockout mice.^22^

**Fig. 2:**
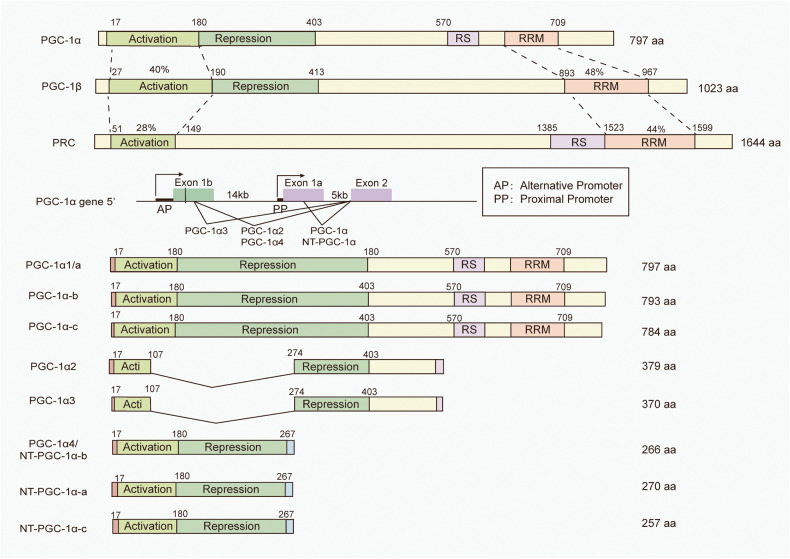
Domain structure of the PGC-1s family and PGC-1α isoforms. The N-terminal region of PGC-1s is a conserved activation domain (AD). Adjacent to the N-terminal region of PGC-1α/β is a domain that represses their own activity, called the RD. The C-terminal region encompasses a well-conserved RRM, which participates in RNA alternative splicing. Moreover, the N-terminal of RRM also plays an important role in mRNA splicing, known as RS domain, which only exists in PGC-1α and PRC, but not in PGC-1β. Moreover, the existence of several promoter regions of a single PGC-1α, along with alternative splicing, leading to the production of PGC-1α isoforms. PGC-1α (also named PGC-1α1or PGC-1α-a) and NT-PGC-1α-a are transcribed by the proximal promoter of PGC-1α gene. Other PGC-1α isoforms are transcribed by a novel exon 1, located 13.7 kb upstream to of the proximal transcription start site

The N- and C-terminal ends of the three members are highly homologous (Fig. 2). The N-terminal region of PGC-1s contains conserved leucine-rich LXXLL motifs and acts as activation domain. This domain is responsible for recruiting histone acetyltransferase proteins, including steroid receptor coactivator (SRC)-1 and cAMP response element-binding (CREB) binding protein/p300.^13^ These histone acetyltransferase proteins facilitate the remodeling of histones within chromatin and further increase the transcriptional activity of PGC-1s. Adjacent to the N-terminal region of PGC-1α/β is a domain that represses their own activity, known as the repression domain (RD). The C-terminal region encompasses a well-conserved RNA recognition motif (RRM), which participates in RNA alternative splicing.^23^ Moreover, the N-terminal of RRM, known as serine/arginine-rich stretch domain, also plays an important role in mRNA splicing. This is unique to PGC-1α and PRC, not found in PGC-1β.^16,24^ Host cell factor (HCF) acts as a coactivator to regulate gene expression during cell cycle progression and enhances the transcriptional activity of PGC-1s.^16^ In addition, the C-terminal region of PGC-1s contains several binding sites for other transcription factors, including forkhead box O (FOXO) 1 and yin yang 1 (YY1).^25,26^ PGC-1s have been demonstrated to co-activate transcription factors, such as PPARs, NRFs, and ERRs, which regulate the expressions of genes implicated in mitochondrial biogenesis, oxidative stress, and energy metabolism.^27–30^ Consequently, PGC-1s are recognized as one of the principal regulators in diverse cellular events.

### Upstream modulators of PGC-1s

**Fig. 3:**
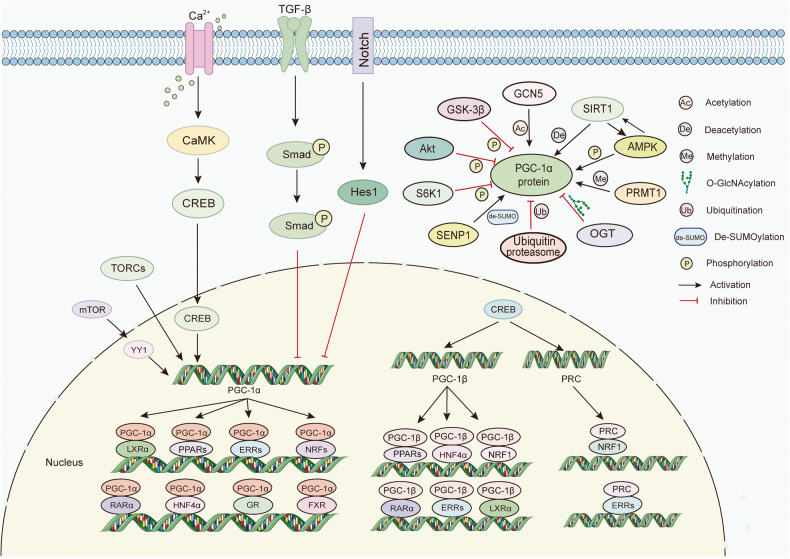
The transcriptional regulatory mechanism and coactivators of the PGC-1s. Upstream modulators, such as YY1, CREB, Smad, Hes1, and TORCs regulate the transcriptional activity and levels. Moreover, PGC-1s play indispensable roles in various cellular events by coactivating transcription factors, including PPARs, ERRs, NRFs, HNFs, LXR, FXR, RARα, and GR. The expressions of PGC-1α are extensively regulated by post-translational alterations. For example, AMPK promotes the activity of PGC-1α by phosphorylation, while Akt, GSK-3β, and S6K1 inhibits PGC-1α by phosphorylation. GCN5 and SIRT1 mediates the deacetylation and acetylation of PGC-1α, respectively. Moreover, GlcNAc transferase (OGT) O-GlcNAcylate PGC-1α, thus protecting it from degradation protein arginine, while PGC-1α can be rapidly degraded in the nucleus through the ubiquitin-proteasome system. PGC-1α is inhibited by SUMOylation, and SENP1 facilitates the activity of PGC-1α through de-SUMOylation. Protein arginine methyl-transferase 1 (PRMT1) methylates PGC-1α, contributing to the induction of endogenous target genes of PGC-1α. These post-translational modifications further broaden the dimensions of the regulatory network and perform critical roles in PGC-1α translocation and activation

Numerous studies have reported that the expression of PGC-1s is extensively regulated by transcriptional and post-translational alterations in response to various external stimuli. For example, exercise enhances a pronounced anti-inflammatory phenotype that visceral adipose tissue possesses during aging, which is linked to the upregulated mRNA levels of PGC-1α.^31^ Protein post-translational modifications, including phosphorylation,^32^ deacetylation,^33^ and methylation,^34^ further broaden the dimensions of the regulatory network and play critical roles in the translocation and activation of PGC-1s. We will concentrate on a couple of upstream modulators, which exert indispensable roles in these modifications, providing a comprehensive and detailed landscape for the regulation of PGC-1s (Fig. 3).

#### CREB and TORC

The transcriptional regulation of PGC-1α is orchestrated predominantly by the critical transcriptional factor CREB activation because the *PGC-1α* gene possesses a well-conserved binding site for CREB. In muscle cells, calcium-signaling components modulate the expression of PGC-1α, in which CREB is a key player. CaMKIV, as the calcium-dependent kinase, activates CREB, which in turn, binds to a conserved cAMP response element in the promoter of PGC-1s.^35^ Herzig et al. elucidated the activation mechanism of gluconeogenic genes during fasting.^14^ Specifically, during prolonged fasting, CREB potentiates gluconeogenic genes including phosphoenolpyruvate carboxykinase (PEPCK), pyruvate carboxylase, and glucose-6-phosphatase (G6P) by increasing the expression of PGC-1 in the liver.^14^ Moreover, during osteoclastogenesis, CREB directly targets PGC-1β, as it binds to the two CRE elements located 5.4 kb and 4.2 kb upstream in the PGC-1β promoter.^36^

Transducers of regulated CREB-binding proteins (TORCs) are generally considered to promote CREB-dependent gene transcription.^37^ Wu et al. screened 10,000 human full-length cDNAs and identified TORCs as upstream regulators of PGC-1α. When TORCs are forcefully expression in primary muscle cells, it induces its downstream target genes involved in the mitochondrial respiratory chain and TCA cycle, which largely depends on PGC-1α.^38^

In summary, CREB and TORCs strongly induce the PGC-1α signaling pathway, linking external signals to the transcriptional program of cellular events.

#### SIRT1 and GCN5

Silent information regulator sirtuin 1 (SIRT1) acts as a cellular sensor to detect energy availability and plays a variety of pivotal roles in cellular biology, such as inflammation, metabolism, oxidative stress, and apoptosis.^39^ As the first identified deacetylases for PGC-1α, SIRT1 requires the coenzyme NAD+ as a substrate for its function and is activated when the amounts of NAD+ or NADH or the NAD + /NADH ratio in cells change.^40^ Once activated, SIRT1 interacts with and deacetylates PGC-1α at specific lysine residues, in a NAD + -dependent manner, further promoting FAO and gluconeogenesis.^33,40^ In the liver, SIRT1 knockdown results in mild hypoglycemia, increased systemic glucose and insulin sensitivity, and decreased glucose generation. On the other hand, overexpression of SIRT1 reverses these changes, relying on the presence of PGC-1α.^41^ Notably, SIRT1 also plays a crucial role in regulating mitochondrial bio-oxidation synthesis in a PGC-1α dependent manner.^42^ PGC-1α and SIRT1 are localized in the mitochondrial matrix in the cytoplasm. The activation of PGC-1α by SIRT1-mediated deacetylation interacts with mitochondrial transcription factor A (TFAM), then enhancing TFAM coactivation and more efficient mitochondrial DNA (mtDNA) transcription. This is accompanied by the augmented activity of nuclear PGC-1α, allowing for the concomitant transcription of nuclear-encoded mitochondrial genes. This supports the idea that PGC-1α and SIRT1 are at the center stage of mitochondrial-nuclear communications.^42^

Considering that deacetylation is a reversible process, it is not surprising that PGC-1α can be mastered through acetylation. GCN5 has been identified to be the specific acetyltransferase for PGC-1α.^43^ GCN5 induces the translocation of PGC-1α to subnuclear domains and represses its function, ultimately repressing PGC-1α-induced gluconeogenic gene expressions and hepatic glucose secretion.^43^ SRC-3 knockout mice exhibit a more favorable metabolic profile compared to wild-type (WT) littermates, which is attributed to enhanced mitochondrial function and energy expenditure following PGC-1α activation. Specifically, SRC-3 enhances the expression of GCN5, thereby facilitating PGC-1α acetylation.^44^ Additionally, PGC-1β can be acetylated by GCN5 on at least 10 lysine residues located throughout the protein. Importantly, GCN5 greatly represses PGC-1β-induced endogenous target genes, including medium chain acyl CoA-dehydrogenase and glucose transporter 4 (GLUT4), further blunting the response to glucose transport induced by PGC-1β, illustrating that the acetylation of PGC-1β by GCN5 plays a crucial role in the modulation of glucose and lipid metabolism.^45^

Therefore, GCN5 and SIRT1 appear to function as a yin-yang pair, responsible for regulating the activity of PGC-1s. Conducting additional research on whether the activity of GCN5 and SIRT1 is also oppositely influenced by internal and external stimuli may contribute to the therapeutic applications of PGC-1s.

#### AMPK

AMP-activated protein kinase (AMPK), a member of the serine/threonine kinase group, serves as the metabolism guardian by participating in sensing the availability of nutrients and energy.^46,47^ When there are changes in energy availability and thus fluctuations in the adenosine triphosphate (ATP)/adenosine diphosphate (ADP) or ATP/adenosine monophosphate (AMP) ratio, AMPK is activated. As a result, activated AMPK restores energy homeostasis by promoting catabolic pathways and restraining anabolic pathways.^48,49^ Importantly, activated AMPK not only increases the transcription of PGC-1α,^50,51^ but also directly phosphorylates PGC-1α protein at threonine-177 and serine-538, ultimately ameliorating mitochondrial function, energy metabolism, and insulin resistance.^32,52^

Interestingly, AMPK modulates the deacetylation of PGC-1α by SIRT1, which explains many convergent biological effects of AMPK and SIRT1 on energy metabolism.^53,54^ During fasting and after exercise, AMPK serves as an initial sensor of energy stress to regulate nicotinamide phosphoribosyl transferase expressions and intracellular NAD+ levels, which in turn affects the activity of SIRT1 on downstream targets such as PGC-1α.^54^ The AMPK activator 5-aminoimidazole-4-carboxamide-1-b-D-riboside (AICAR) significantly increases PGC-1α activity on its own promoter in C2C12 myocytes, but this increment reduces over 60% in SIRT1^-/-^ mouse embryonic fibroblasts. The absence of SIRT1 also compromises AICAR-induced PGC-1α-dependent transcriptional activity on other target genes.^55^

#### Akt

Protein kinase B (PKB, also known as Akt), a conserved serine/threonine kinase member of the AGC family of proteins, is considered to be expressed at the crossroads of multiple cellular processes.^56^ Interestingly, PGC-1α binds and coactivates FOXO1 in a manner that is inhibited by Akt-mediated phosphorylation, thus participating in insulin-regulated hepatic gluconeogenesis.^25^ Moreover, Akt has the ability to stabilize the Cdc-like kinase 2 (Clk2) protein, which phosphorylates the serine-arginine domain of PGC-1α and represses the activity of PGC-1α.^57^ Of note, there are three isoforms of Akt (Akt1, Akt2, and Akt3), which have overlapping and distinct roles and sometimes even perform contrasting functions.^58^ Several studies have explored the roles of Akt isoforms in regulating PGC-1α. Akt2 can directly phosphorylate PGC-1α at Ser 570, which further prevents the recruitment of PGC-1α to the cognate promoters, ultimately inhibiting gluconeogenesis and FAO.^59^ Akt2 ablation initially increases the mitochondrial volume and upregulates PGC-1α.^60^ Wright et al. showed that Akt3 silencing increases the cytoplasmic accumulation of PGC-1α, and reduces the expression of PGC-1α target genes.^61^ They further confirmed that Akt3 blockade increases chromosome maintenance region-1 (CRM-1, a major nuclear export receptor) expression to enhance PGC-1α nuclear export instead of direct effects on post-translational modifications of PGC-1α.^62^ However, Akt1 activation leads to an increment in the expression of PGC-1α, which increases mitochondrial biogenesis and induces apoptosis resistance, further contributing to the pathogenesis of pulmonary fibrosis.^63^ In brief, the different modulation of PGC-1α by Akt isoforms may be due to diverse regulatory levels and cellular processes, and more comprehensive investigation regarding the exact mechanism of Akt isoforms in regulating PGC-1α are required.

#### GSK-3β

Glycogen synthase kinase 3β (GSK-3β) is also a busy serine/threonine kinase, with over 100 known substrates to deal with.^64^ Among these substrates, one of the main targets is PGC-1α.^65,66^ Olson et al. discovered that PGC-1α contains two Cdc4 (the F-box component of the SCF^Cdc4^ ubiquitin ligase) phosphodegrons that bind to Cdc4, which results in SCF^Cdc4^-mediated ubiquitylation and proteasomal degradation of PGC-1α. This process requires GSK3β-dependent phosphorylation at the T295 site.^65^ Interestingly, GSK3β-dependent phosphorylation is also required for nuclear degradation of PGC-1α in response to stress. When exposed to hydrogen peroxide, activated GSK-3β phosphorylates PGC-1α, leading to intranuclear proteasomal degradation, which is also observed in mice both in the oxidative stress response and caloric restriction (CR).^66^

Additionally, in skeletal muscle cells, the inactivation of GSK-3β potently increases the abundance of PGC-1α and oxidative metabolism.^67,68^ Further investigation has confirmed that the inactivation of GSK-3β results in the dephosphorylation of transcription factor EB (TFEB), which then induces the translocation of the TFEB protein to the nuclear. This in turn elevates the activity of the PGC-1α promoter, leading to increased expression and protein abundance of PGC-1α.^69^ Omi is a serine protease present in the mitochondrial space. Under stressful conditions, Omi is released into the cytosol, where it promotes apoptosis through both caspase-dependent and -independent pathways.^70^ The loss of Omi protease activity gives rise to the degradation of PGC-1α, in which GSK-3β is an essential mediator.^71^ Overall, PGC-1α functions as the downstream effector of GSK-3β, enabling GSK-3β to exert an indispensable function in various cellular events.

#### Epigenetic modulatory mechanisms of PGC-1s

Some epigenetic regulations, such as DNA methylation and miRNA regulation, also play an important role in modulating PGC-1s. Wu et al. discovered a growth arrest and DNA damage-inducible β (Gadd45β)-dependent pathway that promotes hepatic glucose production. Mechanistic study revealed that Gadd45β, in conjunction with ten-eleven translocation 1 (TET1), promotes DNA demethylation of the PGC-1α promoter, thereby stimulating PGC-1α expression and promoting gluconeogenesis and hyperglycemia.^72^ In type 2 diabetes mellitus (T2DM) patients, the methylation levels of PGC-1α promoter in skeletal muscle, adipose tissue, and pancreatic islet cells are higher compared to normal individuals.^73,74^ Additionally, PPARGC1A methylated DNA/unmethylated DNA ratio in the liver has a significant correlation with plasma fasting insulin levels and homeostasis model assessment of insulin resistance.^75^ Interestingly, acute endurance exercise can induce the reposition of -1 nucleosome from the transcriptional start site and decreases the methylation level of -260 nucleotide, promoting the transcription of PGC-1α.^76^ These data suggest that DNA demethylation links PGC-1α with metabolic disturbance.

Moreover, several miRNAs have been confirmed to directly target PGC-1α, thus playing crucial roles in various biological processes.^77–83^ For example, the 3’-untranslated region (UTR) of PGC-1α mRNA revealed two conserved miR-23a sites. The activation of miR-23a inhibits gluconeogenesis in hepatocellular carcinoma by decreasing the level of G6P and PGC-1α.^83^ Du et al. found that the suppression of miR-23a restores the PGC-1α/p-dynamin-related protein 1 (Drp1) cascade, which improves mitochondrial membrane potential (MMP) and inhibits oxidative stress and cardiomyocyte apoptosis, thereby improving doxorubicin-induced cardiotoxicity.^78^ Moreover, miR‑696 also play an important role in gluconeogenesis and insulin resistance by downregulating PGC-1α.^84^ A luciferase reporter assay indicated the direct recognition of miR‑696 in a specific location within the 3’-UTR of PGC-1α transcripts.^84^ miR-696 overexpression also impedes mitochondria biogenesis and FAO by inhibiting PGC-1α.^85^ In the future, gaining a comprehensive understanding of miRNA regulation in PGC-1α provides hope for developing miRNA agents targeting PGC-1α.

#### Others

In addition to the main modulators, a diverse set of molecules or modification modes that can effectively regulate the expression and activity of PGC-1s have also been well described.

At the transcription level, Smad3 induced by TGF-β directly binds to the promoter of PGC-1α to decrease the levels of PGC-1α in 3T3-L1 cells, which links TGF-β activity to glucose tolerance and energy homeostasis.^86^ Moreover, HES1, a gene targeted by Notch, is strongly negatively correlated with PGC-1α in human kidney tubule samples. The ChIP assay confirmed direct binding of Hes1 to the promoter region of PGC-1α.^87^ In addition, the mammalian target of rapamycin (mTOR) mediates the interaction between PGC-1α and YY1, leading to an increase in PGC-1α promoter activity.^26^

At the post-translational level, S6 kinase 1 (S6K1) is an identified phosphorylation modulator of PGC-1α. Lustig et al. demonstrated that S6K1 phosphorylates PGC-1α on Ser 568 and Ser 572 within its arginine/serine-rich domain.^88^ Further research has revealed that S6K1-mediated phosphorylation represses the PGC-1α coactivation on hepatocyte nuclear factor (HNF) 4α, thereby significantly impairing the ability of PGC-1α to promote gluconeogenesis in vitro and in vivo.^88^ Besides, protein arginine methyl-transferase 1 (PRMT1) methylates PGC-1α, contributing to the induction of endogenous target genes of PGC-1α.^34^ Moreover, HCF C1 has the capacity to recruit O-GlcNAc transferase (OGT) to O-GlcNAcylate PGC-1α, thus protecting it from degradation and promoting gluconeogenesis.^89^ Rytinki et al. revealed the role of SUMOylation in the regulation of PGC-1α. They found that a lysine residue 183 located in the N-terminal activation domain of PGC-1α undergoes reversible SUMOylation.^90^ The SUMO-specific protease 1 (SENP1) facilitates PGC-1α, which is necessary for the expression of mitochondrial genes and subsequent mitochondrial biogenesis.^91^ As mentioned above, PGC-1α can be rapidly degraded in the nucleus through the ubiquitin-proteasome system.^65,92^ In addition, synoviolin (Syvn)1/Hrd1/Der3, an ER-resident E3 ubiquitin ligase, can trap PGC-1β in the perinuclear region and directly ubiquitinate it, thus impairing energy metabolism.^93^

### Partners and downstream effectors of PGC-1s

As irreplaceable nodal regulators in a variety of physiological processes, PGC-1s coactivate the expression of many partners, as exemplified by PPARs, ERRs, NRFs, HNFs, liver X receptor (LXR), farnesoid X receptor (FXR), retinoic acid receptor α (RARα), and glucocorticoid receptor (GR).^27,94–99^ In this section, we will describe the intimate association between the first four transcription factors and PGC-1s, courtesy of the most intensive research, and others will be shown in the Fig. 3.

#### PPARs

Just like their name suggests, PGC-1s are PPARs-interacting proteins and they synergistically participate in the development of many diseases. PPARs, originally cloned in 1990, belong to the extended nuclear hormone receptor family and consist of three isotypes known as PPARα, PPARβ/δ, and PPARγ, and are mainly expressed in the kidney, liver, small intestine, and heart.^100–103^ PGC-1s have been demonstrated to directly cooperate with PPARs in controlling the transcription of nuclear genes that encode FAO enzymes.^15^ Li and colleagues provided insight into the structural and biochemical basis behind the binding selectivity of PPARγ to PGC-1.^104^ The initial LXXLL motif has the strongest affinity for binding to PPARγ. Specifically, the ligand-binding domain of PPAR is composed of 13 helices and four short strands that are folded into a three-layer helical sandwich and different helix forms a charge-clamp pocket, where the LXXLL motif of PGC-1 is docked.^104^

In many animal models, researchers have emphasized the importance of their synergistic effects. For example, patatin-like phospholipase domain containing protein 2 (an adipose triglyceride lipase, also referred to as Atgl) can generate essential mediators involved in the lipid ligands production for PPARs activation. Atgl deficiency downregulates the mRNA levels of PPARα and PPARδ, which results in the decreased expression of PGC-1α and PGC-1β, followed by the severe disruption of mitochondrial substrate oxidation and respiration in the heart, ultimately causing excessive lipid accumulation, cardiac insufficiency, and lethal cardiomyopathy.^28^ This is in accord with that PPARα is crucial for BAT thermogenesis via induction of PGC-1α during lipid catabolism.^105,106^ Treatment with GW501516, which activates PPARδ, robustly upregulates the mRNA levels of lipid metabolism genes, but this effect is completely abolished when both PGC-1α and PGC-1β are absent.^107^ Apart from the regulation in transcription level, PPARβ modulates PGC-1α in post-translational modification. PPARβ binds to PGC-1α and limits its ubiquitination, which protects PGC-1α from degradation and increases the levels of PGC-1α, thus playing principal roles in the adaptive increase of mitochondrial enzymes in skeletal muscle by exercise.^108^

Meanwhile, PGC-1α performs critical biological functions through a PPARs-dependent pathway. Overexpression of PGC-1α in human epithelial ovarian cancer (OC) cell line Ho-8910 induces apoptosis through the coordinated regulation of Bcl-2 and Bax expression, However, this effect is partially hindered by the PPARγ antagonist GW9662 and suppression of PPARγ.^109^ Additionally, downregulated PGC-1α levels increase the expression of β-secretase, a key enzyme involved in amyloid-β (Aβ) production. However, PGC-1α does not affect Aβ and β-APP cleaving enzyme (BACE1) levels in N2a cells transfected with PPARγ siRNA or in PPARγ knockout fibroblasts.^110^ Intriguingly, PPARβ/δ activator GW501516 can upregulate PPARα levels, PPARα-DNA binding activity, and PPARα-target genes involved in FAO, reflecting the magnification effect of PPARβ in the PGC-1α-PPARα signaling system.^111^ Briefly, the aforementioned results underscore the existence of feedback mechanisms and interaction patterns between PGC-1s and PPARs, which take part in a spectrum of cellular events.

#### ERRs

ERRs are orphan members of the nuclear receptor superfamily and consist of three subtypes including ERRα, ERRβ, and ERRγ.^112^ In 2002, Huss and colleagues completed the identification of ERRα as a PGC-1α interacting partner by using a yeast two-hybrid approach.^113^ They discovered that ERRα binds to PGC-1α through a Leu-rich motif at amino acids 209-213 and utilizes additional LXXLL-containing domains as accessory binding sites rather than the LXXLL motif at amino acid position 142-146 of PGC-1α, which is distinct from that of other nuclear receptors of PGC-1α.^113^ Soon afterward, another team successfully confirmed these findings and the two levels regarding the modulation of ERRα by PGC-1. In one aspect, PGC-1 upregulates the mRNA expressions of ERRα in the heart, kidney, and muscle. In another aspect, PGC-1 interacts physically with ERRα and enables it to activate transcription.^114^

As one of the best-known partners of PGC-1s, ERRs are required for various functions of PGC-1s. These include regulating FAO-related enzyme, osteocalcin gene expression, mitochondrial biogenesis, glucose oxidation, adaptive metabolism response, and insulin sensitivity.^114–124^ For instance, the forced expression of PGC-1α in C2C12 myotubes induces both mRNA and protein expressions of pyruvate dehydrogenase kinase 4 (PDK4, a negative regulator of glucose oxidation), which is achieved by binding to ERRs.^118^ Furthermore, PGC-1α potently induces vascular endothelial growth factor (VEGF) expression and promotes angiogenesis. These findings suggest that PGC-1α coactivates the conserved binding sites of ERRα in the promoter and in a cluster within the first intron of the VEGF gene.^125^ In mice with double deficiency of PGC-1α and PGC-1β, the expression of CDP-diacylglycerol synthase 1 (Cds1, an enzyme that catalyzes the proximal step in cardiolipin biosynthesis) decreases, resulting in phospholipid abnormality. Further experiments have demonstrated that PGC-1α regulates ERRs to activate the transcription of Cds1.^121^ Under normal conditions, overexpression of either PGC-1α or PGC-1β upregulates protein synthesis and myotube diameter in C2C12 myotubes, while the suppression of ERRα weakens this effect.^126^ ERRα is also required for PGC-1β to stimulate carnitine/acylcarnitine translocase in C2C12 cells.^127^ Consistently, Kamei et al. discovered that PGC-1β functions as ERR ligand 1 and activates ERRs. Transgenic mice overexpressing PGC-1β/ERR ligand 1 exhibit increased expression of the medium-chain acyl CoA dehydrogenase, elevated energy expenditure, and resistance to obesity induced by a high-fat diet (HFD) or genetic abnormality. These findings validate that PGC-1β, acting as a protein-ligand of ERR, contributes to the control of energy balance.^128^

In summary, the PGC-1s-ERRs signaling pathway takes part in various essential biological functions. Coincidentally, ERRα has the ability to directly modulate the transcriptional activity of the PPAR and ERRα-mediated activation of FAO enzyme genes relies on the presence of PPAR.^117^ Additionally, ERRγ is implicated in the initial phase of PGC-1α-induced ERRα expression.^129^ These findings reflect complicated modulatory networks existing in different subtypes of the same coactivators of PGC-1s as well as different coactivators of PGC-1s.

#### NRFs

NRFs, composed of NRF-1 and NRF-2, were originally designated as the core promoter binding element for cytochrome c oxidase subunit IV, whereafter it was found to associate with the expression of nuclear genes encoding subunits of the five respiratory complexes, thereby playing key roles in the maintenance of mtDNA and respiratory chain function.^130–133^ Strikingly, Vercauteren et al. revealed that neither PGC-1α nor PRC directly binds to NRF-2 but they exist together in a complex in vivo. This complex formation is mediated by HCF-1, and all three are related to NRF-2-dependent nuclear genes that control the expression of the mitochondrial transcription factors, such as TFB1M and TFB2M.^134–136^ Besides, PGC-1α is activated during exercise and promotes the development of an endurance phenotype through interactions with PPARα, NRF-1, and NRF-2.^137^

#### HNFs

HNFs, categorized into four families, namely HNF1α/β, FOXA1/2/3, HNF4α/γ, and ONECUT1/2, are responsible for regulating genes involved in lipid homeostasis.^138^ The connection between HNFs and PGC-1s is particularly evident in glucose metabolism, lipoprotein metabolism, and response to fasting.^139–143^ For example, PGC-1α stimulates key genes involved in gluconeogenesis, such as PEPCK and G6P, but this ability is lost when HNF4α is absent.^139^ Moreover, the overexpression of PGC-1α also increases the mRNA of apolipoproteins A-IV, C-II, and C-III through a highly conserved HNF4α response element to interact with HNF4α.^144^ These data emphasize the crucial role of the PGC-1α/HNF4α partnership in nutrient metabolism. PGC-1α also plays a significant role in modulating the binding ability of HNF4α in response to cytokine treatment.^145^ While cytokine treatment does not dramatically change the protein levels of HNF4α and PGC-1α, it does reduce the recruitment of PGC-1α to HNF4α-binding sites, in turn downregulating the likelihood of the HNF4α-PGC-1α complex binding to HNF4α-binding sites.^145^

### The roles of PGC-1s in biological functions and physiological processes

#### The effect of PGC-1s in mitochondrial functions

Mitochondria, serving as organelles responsible for energy generation in OXPHOS, are crucial for the activity, function, and viability of eukaryotic cells.^146^ Indeed, mitochondrial dysfunction has become an initiator and propagator in many pathological processes due to its inability to provide the required energy for tissues with eminent energy demand, such as the heart, brain, and muscles.^147–149^ Multiple investigations have established PGC-1s as master mediators in modulating mitochondrial functions. Mitochondrial biogenesis is an extremely intricate process that responds to the energy demand triggered by developmental signals or environmental stressors and new mitochondria are generated from the ones already present.^150^ This process involves the replication of mtDNA, coordinated expression of mitochondrial and nuclear genes, and the import of nuclear-coded mitochondrial proteins into the organelle and turnover.^151^ When activated by the upstream regulators or stressors mentioned earlier, PGC-1α is transferred from the cytoplasm to the nucleus and enhances the expression of NRFs. Subsequently, NRFs promote the transcription and expression of TFAM, which further boosts the transcription and replication of mtDNA and protein synthesis, ultimately leading to the generation of new mitochondria.^2,134,152^ Conversely, PGC-1α mutation impairs the transcription of TFAM, resulting in dysfunctional mtDNA replication.^153^ Simultaneously, the activation of PGC-1α stimulates the transcription of mitochondrial genes involved in respiratory chain complexes.^1,154^

Complementary to the process of mitochondrial biogenesis, mitochondrial quality control is indispensable for maintaining mitochondrial performance and adaptation. The mitochondrial proteins mitofusin (Mfn) 1/2, optic atrophy 1 (Opa1), and Drp1 mediate the fusion of the outer mitochondrial membranes, the fusion of the inner mitochondrial membranes, and the fission of mitochondrial, respectively.^155,156^ Importantly, aside from its well-established roles in mitochondrial biogenesis, PGC-1α also performs important functions in the dynamic properties of mitochondria, including fusion, fission, and degradation, which often orchestrate not only energy metabolism but also complex cell events.^157,158^ PGC-1α directly induces the transcriptional activity of the Mfn2 promoter and acts synergistically with Mfn2. The loss of Mfn2 reduces the stimulatory effect of PGC-1α on MMP, indicating the presence of a regulatory pathway involving PGC-1α and Mfn2.^159,160^ Moreover, PGC-1α overexpression counteracts the decrement in the expression of Mfn1/2 and Opa1.^161,162^ In contrast, the expression of Mfn1/2 is markedly downregulated in the muscle of the PGC-1α/β deficient mice compared to the other groups, accompanied by mitochondrial morphologic abnormalities, structural derangements, and fusion/fission and biogenic defects.^160,163–165^ Exercise training has been shown to reverse the mitochondrial network fragmentation and improve submaximal ADP-stimulated respiration in a PGC-1α-dependent manner.^165^ Emerging evidence also indicated that PGC-1α directly regulates the expression of Drp1 by binding to its promoter.^166,167^ Remarkably, upregulation of PGC-1α simultaneously increases the expression of Mfn2 and Opa1 while inhibiting the expression of Drp1 and fission 1 (Fis1), thus maintaining the balance between mitochondrial fission and fusion.^168^

Mitophagy is an autophagic mechanism that mediates mitochondrial degradation by specifically targeting and eliminating damaged mitochondria.^169^ A variety of studies uncovered the role of PGC-1α in regulating mitophagy. Overexpression of PGC-1α increases lysosomal capacity and indicators of autophagy flux, such as TFEB, LC3B, Beclin, and LAMP1, to maintain mitochondrial homeostasis.^170,171^ Exercise can enhance mitophagy, but this effect is reduced in the absence of PGC-1α.^172^ Furthermore, NRF-1 binds to the classic consensus site in the promoter of Fundc1 (a mitophagy receptor), thus enhancing mitophagy through its interaction with LC3.^173^ The PTEN-induced kinase 1 (PINK1) and Parkin RBR E3 ubiquitin-protein ligase pathway is the most predominant ubiquitination-dependent mitophagy pathway.^174^ Importantly, there is mutual antagonism between the PINK1/Parkin pathway and PGC-1α. PINK1 affects mitochondrial biogenesis by inhibiting the protein expressions of PGC-1α and mtDNA copy number. In turn, PGC-1α represses the protein expressions of PINK1/Parkin and the levels of mitophagy.^175^

As for PGC-1β, it is induced by CREB during osteoclast differentiation, which facilitates mitochondrial biogenesis and increases iron demand.^36^ 3T3-L1 adipocytes overexpressing PGC-1β manifest broader and more ordered mitochondrial cristae, in parallel with elevated mtDNA, Fis1 mRNA expression, and intracellular ATP levels.^176^ In contrast, electron chain capacity, ATP synthesis, and OXPHOS are reduced in PGC-1β knockout mice.^177–179^ Meanwhile, the transcript levels of genes involved in mitochondrial protein import, such as Tomm40l, Timm44, and Timm8a1, and the transcript levels of Mfn2, Opa1, Drp1, and Fis1 are decreased in PGC-1β selectively ablated skeletal myofibers.^180^ These results suggested that PGC-1β is required for normal OXPHOS and mitochondrial function.

**Fig. 4:**
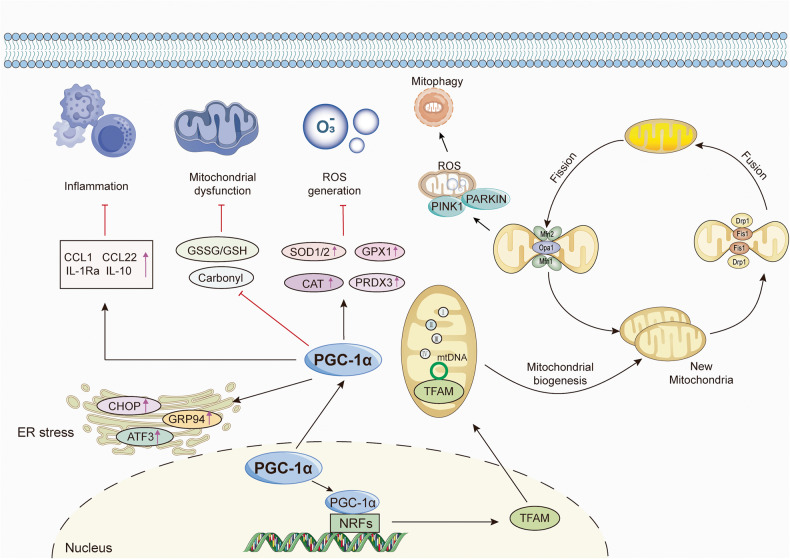
Schematic representation of the critical regulatory roles of PGC-1s in biological functions. PGC-1s, especially PGC-1α, orchestrate the whole processes of mitochondrial life cycle, including mitochondrial biogenesis, fission, fusion, and mitophagy by modulating the coactivators and downstream effectors such as NRFs, Mfn1/2, Opa1, Drp1, and Parkin. PGC-1α fight against oxidative damage by upregulating a wide array of gene expressions regarding anti-oxidant proteins, including SOD, GPX, CAT, and PRDX3. Additionally, PGC-1α and PGC-1β play anti-inflammatory effects by inhibiting the pro-inflammatory factors. Moreover, PGC-1α can improve ER stress by upregulating CHOP, ATF3, and GRP94

Taken together, as irreplaceable nodal regulators in mitochondrial activities, PGC-1α and PGC-1β participate in many vital mitochondrial biological events and establish a multi-link regulatory network based on the control of mitochondrial quality and quantity by regulating downstream effectors (Fig. 4).

#### The effect of PGC-1s in oxidative stress

Oxidative stress refers to an imbalance between the oxidant system and antioxidant defenses caused by the excessive production of ROS or reactive nitrogen species, terminally resulting in damage to DNA, proteins, and cell.^181^ Indeed, PGC-1s also fight against oxidative damage by upregulating a wide array of gene expressions associated with anti-oxidant proteins in different cells, tissues, and organs, including neurons, endothelial cells, retinal pigment epithelium (RPE), and liver.^182–188^

Under metabolic stress, PGC-1α interacts with and coactivates ERG, a fusion oncogene. The PGC-1α-ERG complex then drives the expression of antioxidant genes, including superoxide dismutase (SOD) 1 and thioredoxin (TXN), thus blunting ROS-mediated apoptosis.^188^ PGC-1α^-/-^ retinas exhibit constitutive activation of the VEGF-A signaling pathway, which is partially reversed by antioxidant administration, suggesting that PGC-1α plays a significant role in angiogenesis by regulating ROS homeostasis.^189^ During the maturation of RPE, PGC-1α increases the expression of antioxidant genes, including catalase (CAT), glutathione peroxidase (GPX)1, peroxiredoxin (PRDX) 3, SOD1, SOD2, and TXN2, and represses oxidant-mediated cell death in RPE.^184^ Surprisingly, overexpression of PGC-1α even further inhibits the expression of PGC-1β in RPE. As an example of the transcriptional repression of PGC-1β by PGC-1α, the underlying molecular mechanism is unclear.^184^ In liver steatosis, PGC-1α expression is downregulated. Although hypoxia leads to a remarkable reduction in the expression of antioxidant genes in both PGC-1α^+/+^ and PGC-1α^-/-^ hepatocytes, the restoration of antioxidant protein induced by re-oxygenation is generally diminished in PGC-1^-/-^ hepatocytes, indicating that PGC-1α activity is particularly important in maintaining antioxidant gene expression following organ reperfusion.^190^ Even the loss of a single *PGC-1α* allele exacerbates oxidative stress and hepatic cell death, as shown by the elevated GSSG/GSH ratio and carbonyl content, further diminishing the murine host response to *S. aureus* peritonitis.^191^

Besides, FOXO3a directly regulates many genes that combat oxidative stress in vascular endothelial cells. Importantly, PGC-1α is required for this activity of FOXO3a, as PGC-1α deficiency severely curtails the expression of FOXO3a in endothelial cells.^186^ Friedreich’s ataxia is an autosomal recessive inherited disorder. Marmolino et al. found that PGC-1α and SOD-2 levels are decreased in FRDA cells but do not alter after the addition of hydrogen peroxide. However, PGC-1α siRNA causes a loss of SOD2 response to oxidative stress.^192^ Briefly, these studies revealed that PGC-1s are powerful regulators of ROS metabolism and anti-oxidant enzymes **(**Fig. 4**)**.

#### The effect of PGC-1s in inflammation

Inflammation is an indispensable process that protects against adverse environmental factors by enforcing the defense of homeostasis and the functional and structural integrity of tissues and organs. However, persistent inflammation is regarded as a prime suspect in almost all diseases and underlies a wide range of physiological and pathological processes.^193^ PGC-1α is downregulated by various inflammatory mediators and cytokines.^194,195^ For example, tumor necrosis factor-α (TNF-α) reduces the expression of PGC-1α in the heart through nuclear factor-κB (NF-κB) and p38 mitogen-activated protein kinases (MAPK), leading to a notable enhancement in glucose oxidation rate.^194,196^ Likewise, TNF and interleukin (IL) 1 decrease PGC-1α and PGC-1β, as well as RXR, PPARα, PPARγ, and LXRα, in the liver cells.^197,198^ The similar phenomena also occur in proximal tubule cells, adipocytes, endothelial cells, and oligodendrocytes.^199–202^ Interestingly, NF-κB is constitutively bound to PGC-1α in human cardiac cells, which is further enhanced by TNF-α exposure, eventually giving rise to subsequent dysregulation of glucose oxidation.^203^

Importantly, the activation and upregulation of PGC-1α through genetic or pharmacological manipulation counteract inflammation and play protective roles in different pathological models.^204–207^ NOD-like receptor family-pyrin domain-containing 3 (NLRP3) is an essential sensor in the innate immune system and induces inflammation by promoting the release of the pro-inflammatory cytokines IL-1β and IL-18.^208^ PGC-1α has the ability to restrain the release of mtDNA from the mitochondria into the cytosol, oxidative stress, and increase TNFAIP3 (a negative regulator of NLRP3) to suppress NLRP3 inflammasome.^209^ In addition, both PGC-1α and PGC-1β inhibit p65 phosphorylation and PGC-1β blunts the transcription of p65 and p50 in the basal state, thus constraining inflammatory events in muscle cells.^210,211^ Moreover, PGC-1-dependent alteration of the cytokine profile is observed, featured by an upregulation in the anti-inflammatory factors, including CC chemokine ligand (CCL) 1, CCL22, IL-1Ra, transforming growth factor (TGF-β), and IL-10, and a remarkable inhibition of the pro-inflammatory factor IL-12.^211^ Based on these current studies, PGC-1s contribute to the anti-inflammatory environment in muscle and are important suppressors of inflammation **(**Fig. 4**)**.

#### The effect of PGC-1s in endoplasmic reticulum homeostasis

The endoplasmic reticulum (ER), a complex and dynamic organelle, is responsible for the folding and trafficking of proteins that enter the secretory pathway. When ER functions are dysregulated and overwhelmed, the ER enters a stress state and the highly conserved unfolded protein response (UPR) are activated to restore ER homeostasis.^212,213^ Of note, there are reciprocal regulatory roles between PGC-1α and ER stress **(**Fig. 4**)**. When faced with ER stressors, the mRNA levels of PGC-1α are markedly upregulated.^214^ Importantly, PGC-1α overexpression induces the expression of chaperones, such as BiP and GRP94, and the stress markers like ATF3 and CHOP. However, muscle-specific PGC-1α knockout mice show defective upregulation of ER chaperones and experience exacerbated ER stress after repeated exercise challenges. Mechanistic study has shown that PGC-1α plays an important role in the modulation of the UPR through coactivating ATF6α, a well-characterized sensor in UPR, thus contributing to skeletal muscle adapt to exercise training.^214^ Subsequently, Misra et al. illustrated that ERRγ binds to a responsive element in the ATF6α promoter, which requires the presence of PGC-1α.^215^ In acute kidney injury, overexpression of PGC-1α inhibits ER stress through the UPR pathway, thereby suppressing apoptosis via both the mitochondrial and ER pathways.^216^ Of interest, ER stress can in turn inhibit PGC-1α through suppressing C/EBPβ transcriptional activity, leading to mitochondrial dysfunction and subsequent diabetic embryopathy.^217^ Montori‑Grau et al. also observed that ER stress decreases PGC-1α expression in human myotubes and mouse skeletal muscle.^218^ Therefore, conducting more extensive investigation on PGC-1α and ER may provide novel insights into communications between mitochondria and ER.

#### The effect of PGC-1s in metabolism

Glucose metabolism refers to a series of complex chemical reactions, including glycolysis, aerobic oxidation, glycogen synthesis, and gluconeogenesis, which are necessary to meet the energy requirements of the vital organs.^219^ The roles of PGC-1s in glucose metabolism have been established, particularly in regulating gluconeogenesis and glucose uptake. In response to fasting, the increased synthesis and release of glucagon by pancreatic α cells binds to its receptor on hepatocytes and subsequently triggers the conformational change of G protein. Then, ATP is catalyzed to cAMP, which further binds to each regulatory subunit of protein kinase A (PKA), resulting in the translocation of PKA into the nucleus, finally phosphorylating CREB. The phosphorylated CREB upregulates the expression of PGC-1α. When PGC-1α is activated by CREB and TORCs or coactivates with HNF4α, PEPCK and G6P are increased, and hepatic glucose output is enhanced.^144,220–222^ After food intake, pancreatic β cells synthesize and release insulin that mediates the phosphorylation of Akt, which further triggers the phosphorylation of PGC-1α. The suppression of PGC-1α mediated by Akt results in impaired glucose homeostasis.^59^ PGC-1α also plays an inhibitory role in hepatic insulin resistance in animal models, such as HFD and Ob/Ob mice.^223–225^ Skeletal muscle is a primary site for the utilization of glucose. In skeletal muscle, the electro-transfection or overexpression of PGC-1α upregulates GLUT4 expression and glucose uptake.^226,227^ In addition, PGC-1α also increases FAO and glycogen synthesis and decreases glycolysis and glucose oxidation, thus upregulating muscle glycogen storage.^228,229^ Therefore, PGC-1α overexpression is harmful in the liver, where it facilitates hepatic glucose production. Conversely, it contributes to the oxidation and decrement of glucose in skeletal muscle. Of note, the roles of PGC-1β in glucose metabolism are not consistent with those of PGC-1α. The capacity of PGC-1β to stimulate gluconeogenic genes is relatively low, partially owing to its inability to coactivate with HNF4α and FOXO1.^230^ Nagai et al. confirmed that PGC-1β knockdown reverses hepatic insulin resistance caused by fructose in both basal and insulin-stimulated states.^231^ Therefore, deeper research focusing on the underlying mechanisms regarding the distinct roles between PGC-1α and PGC-1β may provide new insights for the treatment of abnormal glucose metabolism-related diseases.

Another noteworthy effect of PGC-1s is their roles in modulating lipid metabolism. For example, when PGC-1α is overexpressed in murine primary hepatocytes, triglyceride secretion is reduced and FAO is increased to meet energy needs during fasting.^95^ In accordance with this, Huang et al. discovered that PGC-1α stimulates peroxisomal activity and elevates long-chain and very-long-chain FAO in human primary myotubes.^232^ Interestingly, PGC-1α enhances lipogenesis in skeletal muscle.^229,233^ Mechanically, PGC-1α induces and coactivates LXR on the proximal promoter of fatty acid synthase, directly facilitating de novo lipid biosynthesis.^233^ PGC-1α also upregulates the mRNA and protein levels of FITM1/FIT1, which promotes the formation of lipid droplets.^229^ Besides, PGC-1α plays important roles in white adipose tissue browning and thermogenesis.^234–236^ Remarkably, gene expression array profiling revealed that PGC-1β, but not PGC-1α, induces the expression of several genes involved in converting glucose to fatty acid. This results from that PGC-1β interacts with carbohydrate response element binding protein (ChREBP) and binds to the liver-type pyruvate kinase promoter. This highlights the distinct and indispensable roles of PGC-1β in fatty acid synthesis (FAS).^237^ Nevertheless, when exposed to cold, PGC-1β knockout mice develop abnormal hypothermia and hepatic steatosis induced by HFD. Even the compensatory increase in PGC-1α is insufficient to counteract these effects.^238^ In a mouse model with constitutive hepatic activation of PGC-1β, methionine choline-deficient diet-induced hepatic steatosis is ameliorated, primarily relying on the ability of PGC-1β to drive FAO and citrate cycle, and induce triglyceride secretion.^239^ Liver-specific deletion of PGC-1β leads to impaired FAO capacity and mitochondrial dysfunction, giving rise to hepatic steatosis.^240^ The current data suggested that PGC-1β plays dual roles in governing hepatic fatty acid metabolism as it can regulate both FAO and FAS.

Besides, PGC-1α is implicated in amino acids metabolism. Overexpression of PGC-1α in the skeletal muscle increases the expression of enzymes related to branched-chain amino acid (BCAA) metabolism related, such as branched-chain aminotransferase (BCAT) 2 and branched-chain a-keto acid dehydrogenase (BCKDH), which promotes BCAA catabolism and downregulates the levels of BCAA, including valine, leucine, and isoleucine.^241^ Similarly, overexpression of PGC-1α increases BCAA genes and decreases valine levels, while muscle-specific PGC-1α knockout mice manifests downregulated expression of BCAA genes and levels of 3-hydroxyisobutyrate (a catabolic intermediate of valine).^242,243^ Further study has demonstrated that PGC-1α in myotubes stimulates the catabolism of valine to 3-HIB, which then enhances endothelial fatty acid uptake and promotes lipid accumulation in muscle, leading to insulin resistance in mice.^243^ Additionally, during fasting, PGC-1α enhances the promoter activity of alanine aminotransferase 2 (ALT2) in muscle cells in a dose-dependent manner, which facilitates alanine synthesis and secretion.^244^ Patients with T2DM exhibit more aggravating impairments in BCAA catabolism after a glucose load.^242^ These findings may reflect that PGC-1α conducts a cross-regulatory link among the amino acid catabolism, fatty acid metabolism, and glucose levels.

Overall, in light of the pleiotropic effects of PGC-1s in metabolism, especially in glucose and lipid metabolism, which depends on a high degree of specificity in different tissues, decrypting their roles in metabolism guides an approach to design better pharmacological treatment to attenuate metabolic diseases.

### The isoforms of PGC-1α

Among the three founding members of the family mentioned above, PGC-1α has garnered extensive attention since its discovery over 20 years ago. Notably, in addition to the original PGC-1α discussed previously, several studies revealed the existence of several promoter regions of a single PGC-1α, along with alternative splicing, subsequently leading to the production of PGC-1α variants (Fig. 2). While these isoforms share some similarities in structures and overlapping functions, they still have many distinct properties. This section will specifically examine the structural and functional characteristics of PGC-1α variants.

#### PGC-1α-b and PGC-1α-c

In 2008, two novel isoforms of PGC-1α mRNA, named PGC-1α-b and PGC-1α-c, were discovered. Both isoforms are transcribed by a novel exon 1 (exon 1b), located 13.7 kb upstream to the previously reported exon 1 (exon 1a) of the PGC-1α gene. PGC-1α-b and PGC-1α-c are shorter than PGC-1α by four and 13 amino acids, respectively, and differ only in the N-terminal region of the 797 amino acid long murine full-length protein. As for the differences between the PGC-1α-b and PGC-1α-c, they come from the alternative splicing occurring within exon 1b, in which the upstream-splicing site is used for PGC-1α-b, whereas the downstream-splicing site is used for PGC-1α-c.^18^

Importantly, both PGC-1α-b and PGC-1α-c are functional. Specifically, overexpressing either PGC-1α-b or PGC-1α-c increases the expression of genes involved in mitochondrial biosynthesis and FAO. β2-AR agonist injection, endurance exercise, or resistance exercise leads to an increment in PGC-1α-b and PGC-1α-c mRNA in skeletal muscles.^18,245,246^ Interestingly, while a single bout of restricted blood flow exercise increases both PGC-1α-a and PGC-1α-b transcripts, the upregulation in PGC-1α-b is more significant.^247^ A randomized controlled trial revealed that exercise rapidly upregulates the mRNA and protein levels of PGC-1α-b, with the elevated protein occurring before that of total PGC-1α protein, emphasizing PGC-1α-b as the most exercise-responsive PGC-1 isoform.^248^ Additionally, exercise-induced mRNA responses of PGC-1α isoforms (PGC-1α, PGC-1α-b, PGC-1α-c) are intensity dependent.^249^ Yoshioka et al. found that the alternative promoter of the human PGC-1α gene can be activated by CaMKIV and calcineurin A. CaMKIV can recruit CREB to a putative CRE located downstream of the E-box, thereby activating the PGC-1α-b promoter in cultured myoblasts.^250^ These findings suggest a potential molecular basis by which exercise increases isoform-specific PGC-1α mRNA. Evidence from mice overexpressing PGC-1α-b protein in skeletal muscle further supports the notion that increasing PGC-1α-b protein or function is a useful strategy for sedentary subjects to exercise efficiently. PGC-1α-b overexpression promotes mitochondrial biogenesis 4-fold, increases the expression of fatty acid transporters, enhances angiogenesis in skeletal muscle 1.4 to 2.7-fold, and promotes exercise capacity by 35% and peak oxygen uptake by 20%, highlighting the importance of the induction and activation of PGC-1α-b in the adaptation to exercise training.^251^

#### NT-PGC-1α

Zhang et al. reported a novel truncated form of PGC-1α (NT-PGC-1α) composed of 267 amino acids of PGC-1α and 3 additional amino acids from the splicing insert.^252^ It contains the N-terminal domain, which recruits SRC-1 and CREB-binding protein and has the ability to activate transcription and interact with nuclear receptors. However, it loses key domains related to nuclear localization, interaction with other transcription factors, and protein degradation.^252^ Because of the absence of these sequences, NT-PGC-1α is primarily located in the cytosol (90%) under normal conditions. The highest levels of NT-PGC-1α protein expression are observed in the brain, while the liver has the lowest expression, and its expression in BAT and kidney is similar and intermediate between the liver and brain.^252^ NT-PGC-1α can physically interact with both PPARα and PPARγ and even exhibit stronger dependence on ligands compared to PGC-1α.^252^ Similar to PGC-1α, NT-PGC-1α is highly inducible by fasting, cold exposure, and exercise. Additionally, NT-PGC-1α transcript expression in resting muscle accounts for about half of the total PGC-1α expression after acute moderate-intensity exercise.^252,253^

Notably, ectopic expression of NT-PGC-1α in C2C12 myotube cells upregulates myosin heavy chain and GLUT4, promotes the expression of mitochondrial genes (Cyc1, COX5B, and ATP5B), and increases citrate synthase activity.^254^ In addition, NT-PGC-1α interacts with HNF4α and enhances HNF4α-mediated gene transcription, thus inducing gluconeogenesis in primary hepatocytes.^255^ When NT-PGC-1α is selectively expressed in PGC-1α^-/-^ brown adipocytes, nuclear DNA-encoded mitochondrial genes, including TFAM are significantly upregulated, which is even more remarkable than PGC-1α^-/-^ brown adipocytes expressing PGC-1α.^256^ Subsequently, Chang et al. identified the complete repertoire of PGC-1α and NT-PGC-1α target genes in BAT by unbiased genomic approach. Like PGC-1α, NT-PGC-1α targets a broad spectrum of genes related to ubiquitin-dependent protein catabolism, ribonucleoprotein complex biosynthesis, phospholipid biosynthesis, angiogenesis, glycogen metabolism, and autophagy.^257^ Furthermore, NT-PGC-1α overexpression increases the mRNA expression of PPARα-associated genes and suppresses phenylephrine-induced reductions in carnitine palmitoyl transferase 2 (CPT2) and acyl-coenzyme A dehydrogenase-medium chain (Acadm) expression, thereby regulating fatty acid metabolism, increasing extracellular oxygen consumption, and decreasing lipid droplet accumulation in neonatal rat cardiomyocytes.^258^ In contrast, NT‑PGC‑1α deficiency decreases mitochondrial FAO in BAT.^259^ Strikingly, the same group confirmed that NT-PGC-1α deficiency ameliorates HFD-induced obesity by reducing food intake, increasing fecal fat excretion, and decreasing fatty acid uptake in the intestine, adipose tissue, and liver.^260^ Although these results seem contradictory, which may be due to the different regulation in a particular process of fatty acid metabolism by NT-PGC-1α in different tissues, all these highlighted the role of NT-PGC-1α in regulating whole-body lipid homeostasis.

NT-PGC-1α-b and NT-PGC-1α-c are produced during cold exposure through the alternative first exon together with alternative splicing between exons 6 and 7.^261^ Furthermore, they are highly induced by low-, medium-, and high-intensity exercise, AICAR, and clenbuterol.^254^

#### PGC-1α2, PGC-1α3, and PGC-1α4

Using a targeted PCR strategy, PGC-1α2, PGC-1α3, and PGC-1α4 were cloned.^262^ PGC-1α2 and PGC-1α3 have different first exons but share the same remaining exon/intron structure, resulting in a similar domain structure except for discrete N termini at position.^262^ After a series of splicing events common to both PGC-1α2 and PGC-1α3, exons 4-6 and 9-13 are eliminated and exon 8 are spliced to the 3’ UTR of the *PGC-1α* gene, ultimately producing a common stop codon for both transcripts. The resulting proteins, PGC-1α2 and PGC-1α3 (379 and 370 amino acids long, respectively), contain part of the activation domain and repression domain and completely lack all the C-terminal motifs of PGC-1α. PGC-1α4 (which is identical to NT-PGC-1α-b mentioned earlier) possesses the same alternative exon1 with PGC-1α2 and thus the same N terminus. Unlike PGC-1α2 and PGC-1α3, the mRNA of PGC-1α4 contains a 31 nucleotides insertion between exons 6 and 7, therefore producing a premature stop codon. It is predicted to encode 266 amino acids, a protein of 29.1 kDa.^262^ Comparing the gene sets regulated by each PGC-1α isoform, PGC-1α2 and PGC-1α3 form a distinct cluster from PGC-1α4, which shows higher similarities with the genes targeted by PGC-1α. This indicates that the transcriptional activity of the PGC-1α isoforms is dictated by the conservation of the N-terminal activation domain rather than the presence or absence of the RS/RRM motifs.^263^

The researchers also found that cold exposure induces the expression of all PGC-1α variants in BAT.^262^ However, when examining the genes changes driven by different PGC-1α variants, it was discovered that PGC-1α2 and 3 only affect a very small number of genes that overlap with PGC-1α. The expression of PGC-1α4 in myotubes did not affect the regulation of many classic PGC-1α targets, including mitochondrial OXPHOS genes. In contrast, it specifically induces insulin-like growth factor 1 and represses myostatin, thus regulating skeletal muscle size.^262^ In response to the inflammatory signal mediated by TNF-α, PGC-1α4 also has distinct roles compared to PGC-1α1. PGC-1α1 primarily affects genes involved in nutrient metabolism and mitochondrial biology, and decreases the expression of a wide range of inflammatory genes, but it does not prevent hepatocyte death, while PGC-1α4 uniquely increases the expression of anti-apoptotic gene programs and prevent inflammation-mediated apoptosis in hepatocytes.^264^ The expression of PGC-1α4 in vitro and in vivo induces skeletal muscle hypertrophy, while the loss of PGC-1α4 reverses this result. Importantly, transgenic expression of PGC-1α4 in muscle reduces the loss of muscle mass and strength and improves glucose homeostasis during cancer progression, thereby dramatically ameliorating cancer-induced cachexia.^262^ In addition, transgenic expression of PGC-1α4 in skeletal muscle induces VEGF in vivo, whereas the knockdown of PGC-1α4 abrogates the induction of angiogenesis in response to hypoxia.^265^ A recent investigation revealed that PGC-1α4 partially modulates the metabolic benefits of resistance exercise. Overexpressing PGC-1α4 enhances glucose uptake in mouse myotubes and promotes anaerobic glycolysis in a PPARβ- and AMPK-dependent manner.^266^ These studies have unveiled the important function of PGC-1α4 in regulating diverse cellular processes.

In response to resistance exercise, PGC-1α is reduced regardless of the training state.^267^ PGC-1α2 and PGC-1α3 show a similar induction pattern after acute resistance exercise, with the magnitude of the response exacerbated by training. PGC-1α4 is not responsive to acute resistance exercise, but is significantly induced in the trained state.^267^ Nevertheless, Ydfors et al. found that PGC-1α4 is upregulated by both endurance and resistance exercise in human skeletal muscle.^268^ Another study also indicated that acute resistance exercise, either performed alone or 6 h after aerobic exercise, upregulates PGC-1α4.^269^ These two observations suggested that PGC-1α splice variants does not appear to contribute to distinct adaptations to resistance or endurance exercise.^268,269^ Interestingly, in resistance-trained individuals, PGC-1α4 expression following a resistance exercise session has a triphasic pattern: it initially decreases below baseline levels at 45 minutes after exercise, then increases at 3 h post-exercise, and finally decreases below baseline levels again at 48 h post-exercise. Meanwhile, despite the changes in PGC-1α splice variant expression, total PGC-1α expression remains unchanged and then decreases following resistance exercise.^270^ More studies are needed to understand the effects of exercise on inducing different PGC-1α splice variants and the dynamic alteration of PGC-1α variants mRNA expression following exercise.

#### L-PGC-1α and B-PGC-1α

Apart from the alternative promoter located upstream to the original promoter, there is another promoter of PGC-1α gene (termed exon 1 L) in the human liver, which is located within intron 2, is described. The resulting protein, called L-PGC-1α, is identical to PGC-1α except for a deletion of 127 amino acids at the N terminus (encoded by exons 1, 2 and part of 3). The absence of N-terminal region prevents L-PGC-1α from recruiting SRC-1 and CREB-binding protein and interacting with GCN5. However, because of the reservation of C-terminal containing nuclear localization signal, L-PGC-1α is mainly located in the nucleus and coactivates PPARα, PPARγ, and HNF4α.^271^ Therefore, similar to PGC-1α, L-PGC-1α can enhance FAO and mediate hepatic gluconeogenesis by interacting with these coactivators, thus supporting hepatic ATP production in the fasting state.^271^ Besides, Yao et al. demonstrated that HCV infection upregulates both PGC-1α and L-PGC-1α, which further promotes HCV production. Specifically, HCV infection induces ER stress, which upregulates phosphorylated CREB and L-PGC-1α, finally in turn leading to the involvement in the RNA replication and assembly of HCV, eventually promoting HCV production.^272^

The transcription start site of brain-specific PGC-1α isoforms (B-PGC-1α) is located 587 kb upstream of exon 2.^273^ The full-length brain-specific transcripts contain the newly identified exons and reference gene exons 2–13 arranged in a regular order. Importantly, this novel promoter is active in neuronal cell lines, and haplotypes encompassing the novel promoter are more strongly associated with HD age of onset compared to previously described SNPs or haplotypes for the reference locus.^273^

## The role of Pgc-1s in pathophysiological processes and diseases

### PGC-1s in cancers

**Fig. 5:**
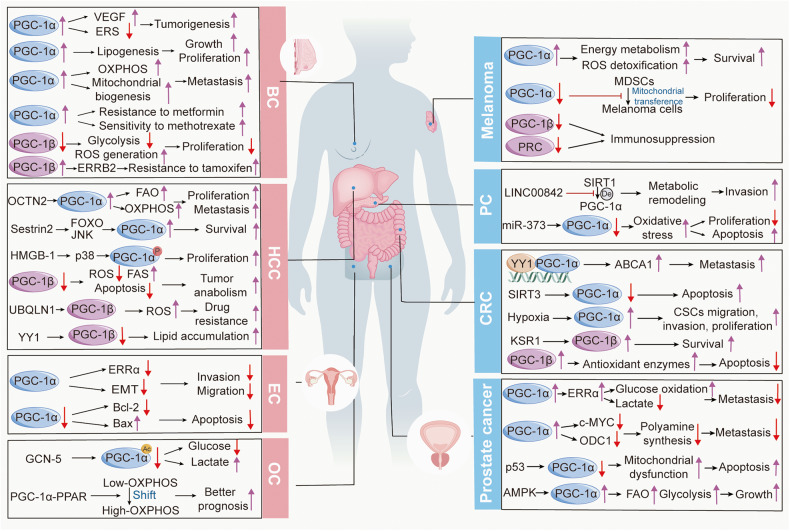
Mechanisms underlying the effects of PGC-1s in various cancers. PGC-1s are widely implicated in imperative cellular events involved in the development of cancers, including mitochondrial OXPHOS, nutrient anabolism and catabolism, autophagy, and apoptosis, and exhibit both detrimental and deleterious effects in cancers

An array of studies suggests that PGC-1s are aberrantly expressed in a diverse range of cancer types and are implicated in tumor proliferation, migration, invasion, metastasis, drug sensibility and resistance, and adaptation to metabolic stress.^274–277^ These findings largely stem from that PGC-1s are irreplaceable central molecules in imperative cellular events involved in the development of cancer, including mitochondrial OXPHOS, nutrient anabolism and catabolism, autophagy, and apoptosis. Noticeably, PGC-1s exhibit different functions not only in distinct types of cancer but also in the same tumor, ranging from antitumor properties to advantageous for cancer cells. These observations imply that the roles of PGC-1s in cancer are both specific to the tissue or organ type and dependent on the particular physiological processes **(**Fig. 5**)**. Therefore, conducting a systematic review to gather current opinions and future exploration to decipher more and deeper mechanisms are extremely significant for solving the therapeutic dilemma.

#### Colorectal cancer

Although colorectal cancer (CRC) was infrequently diagnosed several decades ago, it has been the fourth most deadly cancer in the world, with almost 900, 000 deaths annually nowadays.^278^ Previous research primarily considered PGC-1α as a predictor of lymph node metastasis and poor prognosis in human CRC.^279–281^ Recently, accumulating compelling evidence has emphasized the sophisticated molecule network regarding the roles of PGC-1s in CRC.

In vitro and in vivo studies, PGC-1α knockdown restrains CRC cell proliferation, migration, invasion, and angiogenesis. Mechanistically, PGC-1α interacts with transcription factor YY1, further stimulating ATP-binding cassette transporter 1 (ABCA1) transcription and ABCA1-mediated cholesterol efflux, which aggravates epithelial-mesenchymal transition (EMT), ultimately facilitating CRC metastasis.^282^ Another downstream pathway of PGC-1α in CRC is AKT/GSK-3β/β-catenin.^283,284^ PGC-1α knockdown downregulates the expression of p-AKT, p-GSK-3β, β-catenin, N-cadherin and mitigates cell proliferation, migration, and invasion, while the opposite effects are observed in PGC-1α overexpressing cells.^284^ Moreover, PGC-1α can act as a downstream molecule of SIRT3 in CRC. Under oxidative stress, SIRT3 is recruited with PGC-1α, and suppressing SIRT3 decreases PGC-1α expression, leading to decreased mitochondrial activity and increased apoptosis in cells treated with anticancer drugs.^285^ Cancer stem cells (CSCs), a type of quiescent, pluripotent, self-renewing neoplastic cells, are recognized as tumor-initiating cells.^286^ The researchers discovered that PGC-1α is a master regulator of lactate oxidation and is elevated in normoxic CSCs. Further investigation revealed that PGC-1α mediates OXPHOS, thus promoting metastasis of normoxic colorectal CSCs.^287^ Hypoxia induces PGC-1α expression, which augments mitochondrial biogenesis, OXPHOS, antioxidant enzyme expression, migration, invasion, sphere formation, and proliferation and blocks apoptosis caused by the anti-cancer drug 5-fluorouracil in CRC cells, finally exacerbating tumorigenesis.^288^ Of note, when exposed to 5-fluorouracil, PGC-1α can also promote cancer cell survival via the modulation of mitochondrial function, ER stress, and the apoptotic signaling pathway.^289^

RAS mutations, including HRAS, NRAS, and KRAS, are among the most common oncogenes. The kinase suppressor of Ras 1 (KSR1) is necessary for Ras-induced tumorigenesis. Notably, PGC-1β, as a key downstream effector of KRAS and KSR1, is required for CRC survival both in vitro and in vivo.^290^ The same group further demonstrated that KSR1 protects erythropoietin-producing hepatocellular carcinoma receptor B4 (EPHB4) from lysosome-dependent degradation and increases Myc expression, which upregulates PGC-1β expression to expand the metabolic capacity of the cells and facilitate survival.^291^ Furthermore, overexpressing PGC-1β induces the expression of antioxidant enzymes and renders enterocytes less susceptible to ROS-driven macromolecule damage, thus leading to a delay in apoptosis and an increment in tumor susceptibility and growth rate when exposed to carcinogens.^292^

Collectively, PGC-1s, acting as gatekeepers of redox status and metabolic conditions, play promotive roles in CRC.

#### Hepatocellular carcinoma

Hepatocellular carcinoma (HCC), the fourth most common cause of cancer-related death worldwide, poses a significant global healthcare challenge.^293^ Yang et al. found that organic cation/carnitine transporter 2 (OCTN2) is significantly elevated in HCC and has a strong association with poor prognosis. Mechanistically, the upregulation of OCTN2 promotes the proliferation and migration of HCC cells in vitro and augments the growth and metastasis of HCC, as well as the cancer stem-like properties of HCC by increasing FAO and OXPHOS, which depends on PGC-1α signaling.^294^ When glucose deprivation occurs, sestrin2, a conserved antioxidant and metabolism regulator, stimulates a decrement in intracellular glutamine and PGC-1α levels, leading to a decline in cell survival. Further mechanistic experiments have revealed that sestrin2 forms a complex with c-Jun N-terminal kinase and FOXO1, thereby facilitating the nuclear translocation of FOXO1 and consequently promoting the transcription of PGC-1α.^295^ Additionally, in the diethylnitrosamine-induced HCC model, the genetic blocking of high mobility group box (HMGB)-1 slows tumor cell growth during hypoxia. The researchers further illuminated that HMGB1 translocates from the nucleus to the cytoplasm and binds to cytoplasmic Toll-like receptor, resulting in the activation of p38 and subsequent phosphorylation of PGC-1α, which upregulates mitochondrial biogenesis, finally promoting tumor survival and proliferation.^296^

Unlike PGC-1α, PGC-1β appears to be a double-edged sword in HCC. In one aspect, high level of PGC-1β boosts the expression of ROS scavenger and diminishes ROS accumulation and apoptosis. At the same time, it upregulates the expression of genes involved in FAS and triglyceride synthesis, thus supporting tumor anabolism.^297^ In another aspect, increased degradation of PGC-1β, triggered by UBQLN1, attenuates mitochondrial biogenesis and ROS production in sorafenib-resistant cells under sorafenib treatment, finally causing sorafenib resistance.^298^ Meanwhile, the inhibition of PGC-1β mediated by YY 1 attenuates both medium-chain and long-chain acyl-CoA dehydrogenase levels, leading to the suppression of FAO and exacerbating lipid accumulation, thereby driving HCC progression.^299^ These results reflected that PGC-1s, acting as the downstream targets of some molecules, exert both suppressive and promotive functions in HCC.

#### Breast cancer

Breast cancer (BC) is the most frequent invasive malignancy and the second leading cause of cancer-related deaths in females with an estimated 2.3 million new cases and >685,000 deaths.^300^ Remarkably, although mitochondrial respiration is the main biological function of PGC-1s, additional crucial roles of PGC-1s in glycolysis, glutaminolysis, angiogenesis, and detoxification contribute to its modulatory effects in BC.

Indeed, PGC-1α promotes the growth of ErbB2/Neu-induced mammary tumors by modulating nutrient availability. In vivo, PGC-1α positively regulates the angiogenic factor VEGF and glucose levels and reduces ER stress, thereby alleviating UPR and favoring tumorigenesis.^301^ In addition, glutamine has been reported to play a central role in lipid biosynthesis in cancer cells.^302^ The overexpression of PGC-1α and subsequent activation of ERRα modulates forward and reverses glutamine flux through the citric acid cycle, thereby boosting de novo lipogenesis reactions, particularly in hypoxic conditions, ultimately conferring growth and proliferation advantages to BC cells.^303^ These observations are also supported by the clinical data showing that PGC-1α expression is positively correlated with that of the glutamine pathway in ERBB2+ and high expression of this axis is associated with poor prognosis for BC patients.^303^ BC cells that preferentially metastasize to the lung or bone display relatively high expression of PGC-1α compared to those that metastasize to the liver. PGC-1α promotes BC cell migration and invasion in vitro and augments lung metastasis in vivo, which is linked to enhanced global bioenergetic capacity.^304^ As migratory/invasive cancer cells specifically prefer mitochondrial respiration and increased ATP production, it is not surprising that invasive cancer cells boost OXPHOS, mitochondrial biogenesis, and the oxygen consumption rate by enhancing PGC-1α to perform functional motility of cancer cells and metastasis.^304–306^ This is consistent with clinical analysis that a strong correlation between PGC-1α expression and the formation of distant metastases exists in invasive cancer cells.^305^ In terms of drug response, on the one hand, PGC-1α promotes resistance to metformin (a novel class of potential anti-cancer drugs referred to as energy disruptors) in BC metastasize to the lung cells.^304^ On the other hand, the PGC-1α/ERRα axis results in substantial perturbations in purine biosynthesis and the repression of one-carbon metabolism, which promotes the sensitivity of BC cells and tumors to the anti-folate drug methotrexate.^307^ Therefore, the true roles of PGC-1α in responding to drug therapy in BC remain elusive and require further investigation.

The evidence from the interaction between miRNA and PGC-1α also suggested that PGC-1α plays dual roles in BC. MiR-485-3p and miR-485-5p suppress BC cell metastasis by inhibiting PGC-1α expression. Specifically, overexpression of miR-485-3p and miR-485-5p suppresses mitochondrial respiration and potential for cell migration and invasion in vitro and also abrogates spontaneous metastasis of BC cells in vivo, which are partially relieved by restoration of PGC-1α expression.^308^ In addition, miR‑382 overexpression inhibits tumor‑associated macrophage polarization toward the M2 phenotype and M2‑type cytokine release that promotes EMT and the distant metastasis of BC cells, as well as the ability of tumor‑associated macrophages to promote the malignant behaviors of BC cells, while PGC‑1α expression weakens above changes.^309^ In contrast, miR-217-downregulation increases PGC-1α at both mRNA and protein levels and inhibits BC proliferation and cell-cycle progression, whereas siRNA-mediated PGC-1α downregulation reverses this phenomenon.^79^ Collectively, these observations reflect that PGC-1α plays both deleterious and beneficial roles in BC cell growth, proliferation, migration, and invasion.

Like PGC-1α, the functions of PGC-1β in BC appear to be paradoxical. It has been reported that the inhibition of PGC-1β decreases the glycolytic pathway, increases ROS generation, and impairs cell proliferation.^310^ Similarly, the suppression of PGC‑1β inhibits BC cell growth, proliferation, and migration, and promotes apoptosis by cooperating with the transcription factor FOXA2 or hexokinase domain component 1.^311,312^ Deblois et al. found that ERRα can be recruited to specific sites at chr.17q12 to regulate the expression of ERBB2 in human BC cells and PGC-1β is recruited to ERRα-bound segments in the chr.17q12 amplicon. The ERRα/PGC-1β complex then enhances the development of the ERBB2-positive tumor subtype and tamoxifen resistance in BC through transcriptional control of the ERRB2 amplicon.^313^ Moreover, the overexpression of miR-22-3p restrains the proliferation and migration of BC cells by directly targeting PGC-1β, ultimately regulating the PPARγ pathway in BC.^314^ However, miR-378 fulfils the metabolic shift that TCA cycle activity is reduced and the cells are less dependent on OXPHOS to fulfill their energy demands, which is achieved by suppressing the PGC-1β/ERRγ transcriptional pathway.^315^

Briefly, PGC-1s are of vital importance for BC progression by regulating multiple cellular and physiological processes. However, given the significant impact of BC to worldwide morbidity and mortality and conflictive results, further research is needed to fully comprehend the precise mechanisms underlying the involvement of PGC-1s in BC.

#### Ovarian cancer

OC is the most lethal gynecologic malignancy globally, characterized by poor prognosis and aggressive tumor growth.^316^ The specific molecular for early detection, disease risk stratification, and directing targeted therapies are significant. Research has discovered that PGC-1α/β expressions allow for patient stratification due to their association with the OXPHOS gene program and therefore may be potentially reliable biomarkers predictive of responsiveness to OXPHOS inhibitors in OC.^317^

As previously introduced, GCN5 is responsible for the acetylation of PGC-1α. In cyclin E1-driven OC, GCN-5/PGC-1α signaling is activated and associated with nutrient metabolism. Silencing of GCN5 genetically or pharmaceutically represses the acetylation of PGC-1α, decreases glucose uptake, and increases lactate production.^318^ Interestingly, the metabolomic analyses of frozen high-grade serous OC (HGSOC) samples from the Curie cohort revealed the existence of at least two subgroups with distinct metabolic profiles. High-OXPHOS HGSOC exhibits increased levels of cofactors involved in oxidation-reduction reactions, while low-OXPHOS HGSOC is featured by the accumulation of glutathione metabolism intermediates and choline intermediates. Importantly, PGC-1α-PPAR-mediated mitochondrial biogenesis is sufficient to promote the transition from low-OXPHOS to high-OXPHOS characteristics, which is associated with better prognosis in HGSOC patients. Mechanistically, PGC-1α localizes to subnuclear structures, facilitating its interaction with transcriptional cofactors and coregulators, in which the promyelocytic leukemia (PML) nuclear body constitutes an interface whereby PGC-1α interacts with transcriptional components. All these suggested that the PML protein-PGC-1α axis acts as one of the switches between high- and low-OXPHOS states by modulating the transcription of mitochondrial genes.^319^ In addition, silencing PGC-1α dramatically hinders invasion and migration in cyclin E1-driven OC cell lines.^320^

#### Endometrial cancer

Endometrial cancer (EC) accounts for approximately 76,000 deaths annually among women worldwide, with substantially increased incidence and mortality.^321^ In EC, PGC-1α performs a signaling orchestra with its coactivators, peculiarly ERRs, rather than functioning alone a single player itself. ERRα/PGC-1α overexpression increases the expression of EMT-associated factors including vimentin, Snail, and ZEB1 after exposure to TGF-β and reduces the expression of E-cadherin. However, ERRα knockdown suppresses TGF-β-induced migration and invasion in EC cells.^322^ The mRNA levels of PGC-1α and ERRγ are also positively connected with clinical staging, depth of myometrial invasion, and the number of metastatic lymph nodes in the endometrial adenocarcinomas.^323^ Additionally, the survival of EC cells is dependent on the synergism between PGC-1α and estrogen, which is achieved by the mitochondrial apoptotic pathway.^324^ Specific downregulation of PGC-1α expression promotes apoptosis in HEC-1A cell through the mitochondrial apoptotic pathway by downregulating the expression of Bcl-2 and upregulating the expression of Bax.^325^

#### Melanoma

Melanoma is one of the most common and aggressive skin cancers and continues to be a great contributor to cutaneous cancer-related mortality.^326^ It has been observed that two subpopulations of cells, one expressing high levels of PGC-1α and a second subpopulation with very low PGC-1α expression, exist in melanoma.^327^ Tumors expressing high levels of PGC-1α are associated with lower survival compared to tumors with low PGC-1α expression. Further mechanism research illuminated that mitochondrial energy metabolism and ROS detoxification capacities upregulate in PGC-1α high-expression melanoma cells, which enables melanoma cells to survive under oxidative stress conditions. Conversely, the melanoma cells expressing low PGC-1α levels are more glycolytic and vulnerable to ROS-inducing drugs.^327^ Intriguingly, the heterogeneous expression of PGC-1α within tumors leads to differences in their ability to proliferate or invade. Specifically, the population with low mitochondrial/PGC-1α activity tends to display a pro-metastatic gene program, while the population with high mitochondrial/PGC-1α activity drives a proliferation phenotype. This heterogeneity is critical for melanoma progression through changes in PGC-1α to respond to different signals, including nutrients, and switching between survival-proliferation and invasion-metastasis.^328^ Likewise, Gelato et al. supported the idea that melanoma models with elevated PGC-1α levels are characteristic by a proliferative phenotype.^329^

Amusingly, bone marrow-derived stromal cells (MDSCs) have the capacity to migrate to melanoma tumors. Melanoma proliferation is enhanced by acquiring mitochondria from tumor-supporting MDSCs, while the suppression of PGC-1α reduces mitochondrial transfer from MDSCs to melanoma.^330^ Besides, approximately 30.4-66.0% of cutaneous melanomas are attributed to BRAF mutation.^331^ The researchers illustrated that BRAF activation is associated with decreased oxidative enzymes, diminished mitochondrial quantity and function, and increased production of lactate and BRAF triggers this metabolic reprogramming via the suppression of PGC-1α and MITF, a melanocyte lineage factor.^332^

Noticeably, polymorphism studies revealed that PGC-1β rs32579 polymorphism is linked to tanning ability and provides protection from melanoma.^333^ Another exploration unveils the largely overlooked roles of PGC-1β and PRC in controlling inflammation and immunosuppression in melanoma. The global low expression of PGC-1s increases the expression of immunosuppressive cell surface proteins and cytokines, including galectin-9, PD-L1, PD-L, CD73, and IL-8.^334^ Simultaneously, the expression of PGC-1β and PRC transcripts decreases in tumors that do not respond to anti-PD-L1 therapy and the negative correlation between PGC-1β and PRC with immune genes is strong in the non-responder group. These analyses suggest that reduced expression of PGC-1s in melanoma impairs the response to immunotherapy, possibly through inducing a multigenic immunosuppressive transcription program.^334^

Collectively, these findings indicated that PGC-1s play indispensable roles in melanoma by influencing tumor phenotype, metabolic reprogramming, and immunosuppression.

#### Pancreatic cancer

Pancreatic cancer (PC) is currently one of the most lethal malignancies, with a five-year survival rate as low as 3%.^335^ The function of PGC-1s in PC has drawn extensive attention, mainly focusing on the interaction between PGC-1s and non-coding RNA. LINC00842 (a long intergenic noncoding RNA) has been shown to promote the progression and invasiveness of pancreatic ductal adenocarcinoma (PDAC) by targeting PGC-1α. Specifically, LINC00842 curbs acetylated PGC-1α from deacetylation by SIRT1, resulting in metabolic remodeling of PDAC cells, as exhibited by the transition from cellular mitochondrial oxidative catabolic processes to FAS.^336^ Moreover, miR-373 negatively regulates the expression of SIRT1 by directly binding to its 3’-UTR. Importantly, miR-373 restrains PC cell proliferation but exaggerates apoptosis through modulating oxidative stress response via SIRT1/PGC-1α/NRF2 axis.^337^

Besides, PC stem cells exhibit a distinct metabolic phenotype, which strongly depends on the mitochondrial OXPHOS, whereas non-CSCs mostly require glycolysis. The metabolic phenotype of CSCs is mainly determined by the Myc/PGC-1α ratio.^338^ Considering our current limited knowledge regarding the PGC-1s family in PC, more attention should be paid to elucidating the underlying modulatory mechanisms.

#### Prostate cancer

Prostate cancer remains the most frequently diagnosed non-skin malignancy that affects men’s health and 1 in 25 men globally is diagnosed with this malignant condition during their lifetime.^339^ According to data from the TCGA cohort, several well-established factors, that are associated with prostate cancer progression risks, have been identified, notably PPARGC1A.^340^ It is worth noting that PGC-1s also act as a double-edged sword in prostate cancer.

Some research provides new ideas and evidence supporting the therapeutic targeting of the PGC-1s-ERRs axis in prostate cancer.^341^ PGC-1α expression elicits an obvious decrement in the migratory capacity of PC3 and DU145 cells and a robust anti-invasive phenotype, but ERRα deletion abolishes the induction of target genes of the transcription factor upon induction of PGC-1α.^341^ Equally, PGC-1α activates an ERRα-dependent transcriptional program to control the balance between catabolic and anabolic processes, as shown by the increased glucose oxidation and reduced extracellular lactate levels in PGC-1α expressing cells, thereby exerting a potent anti-metastatic property.^342^ Furthermore, PGC‑1α restrains the metastatic properties of prostate cancer cells by regulating the polyamine biosynthesis pathway. Mechanistically, PGC‑1α inhibits the expression of c-Myc through an ERRα-dependent manner and ornithine decarboxylase 1 (ODC1), the rate-limiting enzyme for polyamine synthesis, further regulating polyamine biosynthesis and prostate cancer aggressiveness.^343^ These results support that PGC-1α-ERRα functions as a tumor-repressive transcriptional complex through modulating metabolic events. p53 is a tumor suppressor gene with extensive and powerful functions, known as the “guardian of the genome”.^344^ Li et al. found that p53 downregulates the expression and nuclear localization of the PGC‑1α protein and stimulates mitochondrial dysfunction, which promotes apoptosis, highlighting PGC‑1α as an essential target of p53-induced apoptosis in prostate cancer cells.^345^

Nevertheless, in contrast to these, the PGC-1s pathway has been demonstrated to promote prostate cancer cell growth.^346,347^ On the one hand, PGC-1α interacts with the N-terminal domain of androgen receptor (AR), participates in the N- and C-terminal interaction of AR, and upregulates the DNA-binding ability of AR to androgen-responsive elements in the prostate-specific antigen enhancer and promoter regions to increase the transcription of AR target genes, finally facilitating prostate cancer cell growth.^346^ On the other hand, prostate cancer cells respond to androgen treatment by increasing glycolysis rates, glucose, and FAO, which is dependent on androgen-mediated AMPK activity and subsequent PGC-1α activation. In other words, androgens regulate prostate cancer cell growth via an AMPK-PGC-1α-mediated metabolic switch.^347^

### PGC-1s in noncancer diseases

#### PGC-1s in cardiac diseases and cardiovascular diseases

**Fig. 6:**
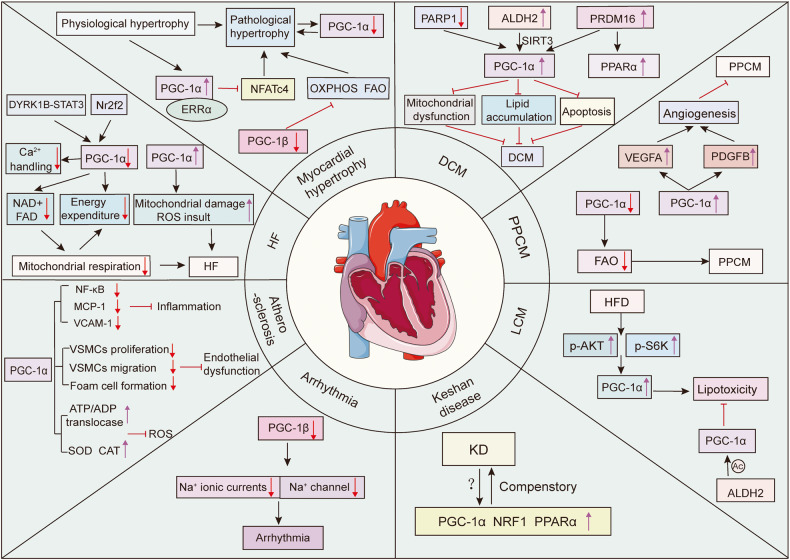
The roles of PGC-1s in cardiac diseases and cardiovascular diseases. (1) In HF, PGC-1α deficiency causes major alterations in mitochondrial respiration and growth, ultimately giving rise to cardiac dysfunction. (2) The expression and activity of PGC-1α initially increase to meet the energy requirements during physiological hypertrophy, but consistently elevated levels of PGC-1α further leads to pathological hypertrophy. Meanwhile, PGC-1β deficiency aggravates the transition from hypertrophy to HF. (3) The upregulation of PGC-1α induced by several upstream molecules restrains DCM development by mediating lipid metabolism, mitochondrial function, and apoptosis. (4) PGC-1α can affect PPCM in three ways: 1) triggering the pro-vascular VEGF-mediated angiogenic signaling; 2) meeting the need for a fuel shift towards FAO；and 3) regulating energy metabolism. (5) HFD-induced reduction in PGC-1 provokes cardiac lipotoxicity. In contrast, PGC-1 overexpression counteracts the fat accumulation and heart defects induced by HFD. (6) The mRNA levels of PGC-1α, NRF1, and PPARα shows compensatory increase in KD, but precise regulatory molecular mechanisms of PGC-1s in KD is unkown. (7) PGC-1β deficiency leads to aberrant Na^+^ ionic currents and Na^+^ channel, then enhancing arrhythmic ventricular phenotype. (8) The mechanisms that PGC-1α represses atherosclerotic disease progression involves in inhibiting ROS, endothelial dysfunction, and inflammation

The connection between the PGC-1s pathway and the cardiovascular system has been investigated since it was discovered. As early as 2000, Lehman et al. identified PGC-1 as an essential regulatory molecule in the control of cardiac mitochondrial number and function in response to energy demands.^3^ Subsequently, a series of studies revealed that PGC-1s play indispensable roles in mediating cardiac fuel transport and consumption, energy state, and the development and function of the heart.^348–352^ For example, PGC-1α expression in the heart significantly increases at birth, which is required for a high-level expression of nuclear and mitochondrial-encoded genes involved in mitochondrial energy transduction and OXPHOS, and for full respiratory capacity.^160^ Therefore, dysregulation of the PGC-1s pathway substantially disrupts cardiac metabolism homeostasis and results in different types of cardiac diseases and cardiovascular diseases **(**Fig. 6**)**.

##### PGC-1s in heart failure

Heart failure (HF), the most devastating consequence of cardiovascular disease, is characterized by variable durations of symptomatic stability even worsening symptoms despite continued therapy.^353^ A variety of research indicated that the mRNA and protein levels of PGC-1s and its coactivator as well as the target genes are downregulated in HF patients.^354–356^ Notably, serum PGC-1α is inversely correlated with energy expenditure and PGC-1α level reflects the degree of myocardial energy expenditure and the systolic function of the left ventricle in patients with chronic HF.^357^ In a cohort of 35 consecutive stable HF patients with severe aortic stenosis who underwent an elective aortic valve replacement surgery, a higher systemic PGC-1α expression is associated with higher SIRT1 levels and Trolox concentration, suggesting a better antioxidant status in these patients. Therefore, PGC-1α can be used as prognostic indicator in cardiovascular diseases.^358^ However, different study groups detected unchanged protein levels of PGC-1α in HF.^359,360^ These contradictory results might be explained by differences in the time point tested and sample diversity. Defining the complete mapping of expression changes of PGC-1α during the whole progression of HF will contribute to more precise therapy.

Some convincing evidence from genetic deletion animal models further supports the critical roles of PGC-1α in HF. For instance, PGC-1α^-/-^ mice display profound cardiac dysfunction in response to cardiac duress, as initiated by constriction of the transverse aorta.^361^ The metabolome analysis revealed that heart-specific knockout of PGC-1α leads to major alterations in the metabolic processes associated with mitochondrial respiration and growth, as demonstrated by the reduced levels of acetyl-CoA, NAD + , FAD, acylcarnitine, and succinic acid, eventually causing HF.^362^ In addition, PGC-1α dysregulation abrogates the recruitment of RNA Polymerase II to metabolic gene promoters, thus inducing HF phenotypes.^359^ Likewise, Naumenko et al. observed that PGC-1α deficient mice develop dilated HF associated with suppression of energy metabolism, compromised calcium handling of cardiomyocytes, and remodeling of electrophysiological properties of cardiomyocytes. Interestingly, they further found more rapid and drastic contractile dysfunction and earlier death in female mice compared with male, suggesting that maintenance of normal phenotype and function are more reliant on intact energy metabolism in female than male hearts.^363^ In addition, PGC-1α also mediates the protective role of nuclear receptor subfamily 2-group F-member 2(Nr2f2) and DYRK1B deletion, validating the potential possibility of targeting PGC-1α for HF therapy.^364,365^

Nevertheless, several other studies manifest that the excessive expression of PGC-1α does not exert a beneficial role and even facilitates the development of HF. Karamanlidis et al. used a transgenic mouse model of moderate overexpression of PGC-1α ( ~ 3-fold) in the heart and found that PGC-1α upregulation does not improve cardiac energetics and function. Long-term overexpression of PGC-1α renders mice more vulnerable to acute cardiac stress and mice fails to protect against cardiac dysfunction caused by chronic pressure overload.^366^ In addition, cardiac-specific overexpression of PGC-1α ameliorates mitochondrial and cardiac function in 3-month-old WT mice but facilitates cardiac aging and markedly shortens lifespan in 12-month-old WT mice due to increased mitochondrial damage and ROS insult.^367^ In summary, owing to the complexity of the signaling pathway and the importance of maintaining cardiac homeostasis, it is necessary to carefully consider and explore the range and period of regulating PGC-1α levels.

##### PGC-1s in myocardial hypertrophy

Myocardial hypertrophy is an adaptive response to physiological and pathological overload. When exposed to overload, activated intracellular hypertrophic signaling pathways facilitate myocardial angiogenesis to dissolve the hypoxic situation and to maintain cardiac contractile function, but sustained overload induces pathological hypertrophy, generally progressing to HF.^368,369^ Growing compelling evidence suggested that PGC-1α is a multifaceted regulator in both physiological and pathological forms of myocardial hypertrophy. Under physiological conditions of increased energy demand, including exercise and fetal heart development, the elevated level of PGC-1α promotes mitochondrial biogenesis and ameliorates energy metabolism.^370,371^ In contrast to this, during pathological myocardial hypertrophy, the expression of PGC-1α is downregulated, which is also associated with a net loss of mitochondrial protein and oxidative capacity.^372,373^

In triiodothyronine (T3) induced cardiac hypertrophy, the mRNA level of PGC-1α decreases first and subsequently increases, but the overexpression of PGC-1α improves cardiac function through increasing energy production and mitochondrial biogenesis. Thus, it is possible that PGC-1α increases via an indirect or compensated mechanism.^374^ Liu et al. revealed the protective mechanisms of PGC-1α on myocardial hypertrophy. PGC-1α represses the expression of calcineurin-nuclear factor of activated T cells c4 (NFATc4) that participates in the regulation of heart development and bioenergetics, prevents its dephosphorylation and nuclear translocation, and further abrogates its binding activity to brain natriuretic peptide promoter, ultimately protecting cardiomyocytes from hypertrophy.^375^ In addition, the injection of AAV9-anti-miR-199a tough decoys virus alleviates cardiac hypertrophy and restores cardiac function, which depends on the PGC-1α/ERRα axis.^77^ Noticeably, a recent investigation demonstrated that PGC-1α expression in the physiological range in pressure overload hypertrophy (POH) preferentially preserves angiogenesis but is not sufficient to prevent POH-induced mitochondrial or contractile dysfunction.^376^ Collectively, facilitating PGC-1α signaling plays a cardioprotective role against pathological myocardial hypertrophy.

As for another member, PGC-1β expression is also diminished in POH. In the transverse aortic constriction model, PGC-1β deficiency aggravates oxidative stress, decreases cardiac efficiency, glucose metabolism, and hexokinase II protein, further accelerating the transition to HF, while PGC-1β activation mediates the protective roles of melatonin and attenuates cardiac contractile function.^377,378^ Considering that there are few studies on PGC-1β or PRC in cardiac hypertrophy and fibrosis, further research is needed in the future.

##### PGC-1s in cardiomyopathy

Cardiomyopathy refers to cardiac dysfunction caused by various factors, such as diabetes, pregnancy, and obesity.^379–381^ This section discusses how PGC-1α plays a vital role in these different types of cardiomyopathy.

Diabetic cardiomyopathy (DCM), resulting from insulin resistance, T2DM, and associated hyperinsulinemia independent of hypertension and coronary heart disease, is a major cause of morbidity and mortality in developed nations.^382,383^ Recent studies have suggested that PGC-1α and its coactivators play regulatory roles in DCM development by mediating lipid metabolism, mitochondrial function, antioxidant defense, and insulin resistance.^384–386^ Mitochondrial aldehyde dehydrogenase (ALDH) 2 serves as an imperative cardioprotective molecule against insulin resistance-induced cardiomyopathy, which is closely linked to the promotion of the SIRT3-dependent PGC-1α deacetylation.^387^ The transcription factor PR-domain containing 16 (PRDM16) is another protective factor in DCM. PRDM16 cardiac-specific deficiency mice manifest worsened cardiac dysfunction, aggravated mitochondrial dysfunction, cardiac lipid accumulation, and apoptosis. Co-IP and luciferase assays confirmed that PRDM16 regulates the transcriptional activity, expression, and interaction of PPARα and PGC-1α, while the overexpression of PPARα and PGC-1α reverses PRDM16 deficiency-induced cellular dysfunction in T2DM model. All these suggested the critical effects of PPARα and PGC-1α in PRDM16-mediated cardioprotective action.^386^ Besides, in the development of DCM, PGC-1α activation is responsible for reversing the Warburg effect to aerobic respiration when exercising, thus enhancing mitochondrial metabolism and energy homeostasis.^388^

Peripartum cardiomyopathy (PPCM) occurs globally and is accompanied by systolic dysfunction that presents in late pregnancy or, more commonly, the early postpartum period.^389^ Mice lacking cardiac PGC-1α develop profound PPCM, as shown by enlarged left ventricular end-diastolic dimensions and left ventricular end-systolic dimensions, and depressed cardiac contractile function.^390^ However, overexpression of PGC-1α in neonatal rat ventricular myocytes (NRVMs) strongly increases angiogenic genes involved in the activation and recruitment of endothelial cells (including VEGFA) and mural cells (including PDGFB), as well as genes that take part in the mitochondrial respiratory chain (including Cycs and Cox5b), suggesting that PGC-1α controls an angiogenic program, which entirely rescues PPCM.^390^ Conversely, β1-Adrenoceptor antibodies-treated postpartum rats manifest PPCM, which is associated with the repression of PGC-1α in parallel with the decline of its downstream transcript VEGF.^391^ Garcia and colleagues have found that methyl donor deficiency aggravates the metabolic condition of PPCM. Specifically, the methyl donor deficiency leads to imbalanced methylation/acetylation of PGC-1α and decreased expression of PPARα and ERRα, further causing detrimental effects on FAO and energy metabolism.^392^ In addition, PGC-1α and its coactivated partners PPARs play principal roles in the regulation of FAO as discussed above.^28,393^ Because of an increasing fuel shift towards high reliance on FAO in the gestational heart,^394^ aberrant FAO can contribute to PPCM. Generally, PGC-1α can affect PPCM in three ways: 1) triggering the pro-vascular VEGF-mediated angiogenic signaling; 2) meeting the need for a fuel shift towards FAO; and 3) regulating energy metabolism.

As previously introduced, PGC-1α plays an important role in regulating lipid metabolism. Therefore, it has a close connection with obesity cardiomyopathy and lipotoxic cardiomyopathy (LCM). HFD intake induces weight gain, hypertrophy and interstitial fibrosis, contractile dysfunction, mitochondrial injury, and apoptosis, whereas ALDH2 offers protection against HFD-induced cardiomyopathy through reversing the changes in CaMKII, SIRT1, and PGC-1α acetylation.^395^ In line with this, HFD-induced reduction in PGC-1/spargel (srl) expression provokes cardiac lipotoxicity. HFD feeding activates TOR signaling (increased p-AKT and p-S6K), which in turn gives rise to the downregulation of PGC-1/srl expression. In contrast, PGC-1/srl overexpression counteracts both the fat accumulation and heart defects induced by HFD. These findings identified an integrated genetic network for counteracting obesity and associated cardiac lipotoxicity, in which PGC-1 is both necessary and sufficient.^396^

In addition, mitochondrial-related gene expression profiles reflect important roles of PGC-1α in the compensatory mechanism of Keshan disease (KD), an endemic dilated cardiomyopathy with unclear etiology. The researchers found that six nuclear receptor-related pathways and eight genes, as well as four energy production-related pathways and five genes are upregulated in KD and PGC-1α-induced energy production plays an important role in the compensatory mechanism of KD.^397^ Recently, Jiang et al. discovered that the mRNA levels of PGC-1α, NRF1, and PPARα are higher in patients with KD. Notably, the area under the curve for the “lactate dehydrogenase (LDH) + PPARα” combination was 0.984, with 96.7% sensitivity and 93.0% specificity, indicating that the combined detection of LDH and PPARα can be performed to diagnose chronic KD.^398^ Nevertheless, our current knowledge of PGC-1s in KD and precise molecular mechanisms is incomplete, and additional work is needed in the future.

##### PGC-1s in arrhythmia

Different from other diseases, PGC-1β rather than PGC-1α has drawn widespread attention in arrhythmia. PGC-1β deficient mice show pro-arrhythmic ventricular phenotype secondary to mitochondrial dysfunction.^399–401^ In addition, cardiomyocyte Na^+^ ionic currents in the age-dependent murine PGC-1β model of ventricular arrhythmia are reduced.^402^ Compared to WT, the protein expressions of the Na^+^ channel in murine PGC-1^-/-^ atria are also reduced.^403^ These changes suggest potential roles of PGC-1β in cardiac electrophysiology and ion channel changes. However, compared to research on PGC-1s in other cardiac diseases, the current understanding of PGC-1s in arrhythmia is only at the tip of the iceberg and is far from adequately sufficient to describe the specific role of PGC-1s in arrhythmia. Thus, further efforts are warranted to fully elucidate PGC-1s involved in the pathologic mechanisms of arrhythmia.

##### PGC-1s in atherosclerosis

Atherosclerosis is a chronic inflammatory and lipid-depository disease of the arterial wall and is a leading cause of acute cardiovascular events and death worldwide.^404^ One case-control survey reported that Gly482Ser polymorphism in the gene encoding PGC-1α contributes to the risk of coronary artery disease.^405^ Meanwhile, the PGC-1α protein is markedly downregulated in human atherosclerotic vessel samples.^80^ These remind the potential effects of PGC-1α in atherosclerosis. Next, we further describe the crucial roles of PGC-1s in inflammation, oxidative stress, endothelial cell dysfunction, and vascular smooth muscle cells (VSMCs) activities during atherosclerosis.

ROS production is the main cause of endothelial cell injury as ROS increase endothelial permeability, promote leukocyte adhesion, and change endothelial gene expression.^406^ Indeed, the powerful induction of PGC-1α in antioxidant proteins greatly contributes to its action in atherosclerosis. TNF-α, a major proinflammatory factor in vascular inflammation, increases intracellular ROS production. Overexpression of PGC-1α in human aortic smooth (HASMCs) and endothelial cells (HAECs) reverses the above phenomenon and suppresses NF-κB activity, and monocyte chemoattractant protein-1 (MCP-1) and vascular cellular adhesion molecule-1 (VCAM-1) expression induced by TNF-α, thus preventing the development of atherosclerosis.^407^ In addition, PGC-1α can enhance ATP/ADP translocase activity and nicotinamide adenine dinucleotide phosphate (NADPH) oxidase degradation through proteasome degradation pathway, further restraining ROS generation and apoptosis in endothelial cells.^408,409^ C1q/TNF-related protein-9 ameliorates oxidized low-density lipoprotein (ox-LDL)-induced endothelial dysfunction, which is mediated by PGC-1α/AMPK-induced antioxidant enzyme.^410^

It is known that VSMCs proliferation is detrimental throughout atherosclerosis.^411^ Accumulating evidence highlighted that PGC-1α protects VSMCs from proliferation, migration, and inflammation.^412–414^ For example, free fatty acids, including oleic acid and palmitic acid, stimulate VSMCs proliferation and migration and result in the formation of organized atherosclerotic plaque. PGC-1α overexpression blocks VSMCs proliferation and migration due to its capacity to prevent ERK phosphorylation, while the suppression of PGC-1α by siRNA enhances the effects of oleic acid and palmitic acid.^415,416^ The regulator of lipid metabolism perilipin 5 (Plin5) knockdown leads to accelerated neointima hyperplasia, excessive proliferation, and migration of VSMCs and inhibits the interaction between plin5 and PGC-1α. Importantly, researchers further illustrated that overexpression of PGC-1α suppresses ROS generation, proliferation, and migration in VSMCs.^417^ The process by which monocytes differentiate into macrophages and macrophages recognize and take up highly ox-LDL particles, which can lead to foam cell formation, is considered one of the vicious points, finally causing atherosclerotic plaque.^418^ Notably, PGC-1α is localized to macrophage/foam cells in the murine aorta where its expression is increased when conjugated linoleic acid attenuates murine atherosclerosis. Overexpression of PGC-1α in bone marrow-derived macrophages diminishes foam cell formation, whereas macrophage-specific deletion of PGC-1α accelerates atherosclerosis in the LDLR^-/-^ mouse.^419^ Methyl-transferase-like 3 (METTL3) acts during ox-LDL-induced monocyte inflammation. Mechanistically, METTL3 and YTH N6-methyladenosine RNA binding protein 2 cooperatively modify PGC-1α mRNA, regulate PGC-1α degradation, and downregulate PGC-1α protein levels, thereby enhancing the inflammatory response.^420^

Aging is considered an independent risk factor for human atherosclerosis and vascular senescence facilitates plaque vulnerability, which greatly increases the possibility of cardiovascular events.^421,422^ Xiong et al. identified PGC-1α as a negative regulator of vascular senescence in vivo and in vitro. Angiotensin II leads to SIRT1 and CAT downregulation and vascular senescence, which is achieved by inducing prolonged lysine acetylation of PGC-1α and interrupting the PGC-1α-FOXO1-SIRT1 feed-forward.^419^ PGC-1α deficiency can also mediate impaired autophagy caused by the downregulation of SQSTM1 (autophagy receptor), thus accelerating vascular aging and atherosclerosis.^423^

In conclusion, the roles of PGC-1α in atherosclerosis have gained extensive attention. PGC-1α deficiency in endothelial cells, VSMCs, and monocytes/macrophages promotes atherosclerosis. Hence, PGC-1α might be a potential therapeutic target for the treatment of atherosclerosis.

#### PGC-1s in neurological disorders

**Fig. 7:**
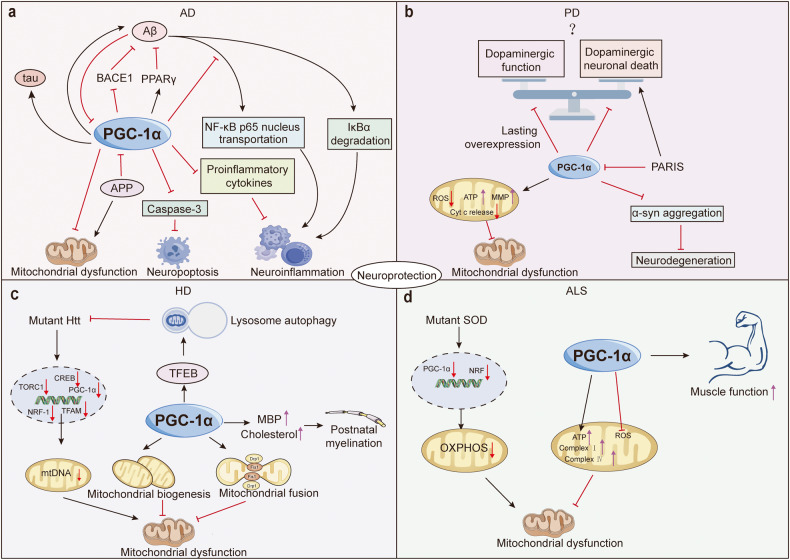
Mechanisms of neuroprotection mediated by the PGC-1α signaling network. **a** PGC-1α inhibits Aβ deposition, neuroinflammation, neuropoptosis, and mitochondrial dysfunction, but it also exacerbates Aβ and tau accumulation in AD. **b** PGC-1α overexpression represses dopaminergic neuronal loss, behavioral deficits, mitochondrial dysfunction, and neurodegeneration, while its lasting overexpression suppresses dopaminergic function in PD. **c** PGC-1α upregulation promotes HTT protein elimination and postnatal myelination, and inhibits mitochondrial dysfunction. **d** PGC-1α upregulation increases ATP production and enhances muscle function in ALS

Neurological disorders, especially neurodegenerative diseases (NDs), are characterized by progressively structural and functional loss of neurons in discrete areas of the central nervous system (CNS), accompanied by memory difficulty, uncontrolled motor activities, and impairment in expressive speech, visuospatial processing, and executive functions, posing looming dire economic and societal impacts.^424^ The more common NDs in the elderly population are Alzheimer’s disease (AD), Parkinson’s disease (PD), Huntington’s disease (HD), and amyotrophic lateral sclerosis (ALS). Given the high metabolic demand of the brain, and the importance of ATP synthesis and the maintenance of mitochondrial function for neuronal activity, PGC-1s have been extensively studied as a center in the network of energy metabolism. Analysis of PGC-1α expression patterns showed that PGC-1α is abundantly expressed in the brain areas, such as the cerebral cortex, hippocampus, striatum, thalamic nucleus, and substantia nigra.^425^ PGC-1α is also implicated in maintaining cholinergic,^426^ glutamatergic,^427^ dopaminergic,^428–430^ and GABAergic synapses.^431,432^ PGC-1α deficiency in specific brain areas, including GABAergic neurons causes short-term habituation, hyperactivity, and exaggerated startle reactivity.^433^ On the other hand, activation or overexpression of PGC-1α can counteract neurological disorders by improving mitochondrial function, neuronal maintenance, neuroinflammation, and protein clearance.^434–436^ Herein, we will provide a complete picture of the role of PGC-1s in different models covering AD, HD, PD, and ALS (Fig. 7), aiding in the design of future studies and advancing investigations of PGC-1α as a therapeutic target in the nervous system diseases treatments.

##### PGC-1s in AD

AD, featured by progressive impairment in cognition, emotion, language, and memory in older population, is an irreversible, multifactorial, and age-related neurodegenerative disease.^437^ A putatively fatal etiological hypothesis is the accumulation of Aβ.^438^ Importantly, there are complicated and direct links between PGC-1α and Aß. Amyloid precursor protein (APP)/PS-1 transgenic mice are popular animal models of AD. BACE1 is the main enzyme involved in Aβ generation. Four months after injection of PGC-1α in APP23 mice, improved spatial and recognition memory concomitant with a significant reduction in Aβ deposition and decreased expression in BACE1 are observed.^439^ The findings by other teams that PGC-1α activation or overexpression severely diminishes the protein expression of BACE1 and Aβ plaques also support the results.^440–442^ In addition, PGC-1α blocks Aβ generation through a PPARγ-dependent mechanism.^110^

Beyond Aβ deposition, emerging evidence strongly suggested that neuroinflammation and mitochondrial dysfunction are prerequisites for AD pathogenesis.^443,444^ Sheng and colleagues showed that expression levels of PGC-1α, NRF-1, and NRF-2 are significantly decreased in both AD hippocampal tissues and APPswe M17 cells. Overexpression of PGC-1α completely rescues, while knockdown of PGC-1α exacerbates impaired mitochondrial biogenesis and deficits in APP mutant M17 cells.^445^ Interestingly, the mRNA expression levels of CREB, PGC-1α, NRF-1, NRF-2, and TFAM are decreased as early as 1 month of age when there is no significant Aβ oligomer deposition in 3xTg-AD mouse (harboring PS1, APP, and tau human transgenes). At later ages, the protein expression of complex II, III, and IV and the activity of complex IV downregulate. These suggest that mitochondrial biogenesis is likely impaired in the ages preceding the development of AD pathology and is related to mitochondrial dysfunction at later ages.^446^ In addition, overexpression of PGC-1α remarkably reduces the level of pro-inflammatory cytokines and dampens the transportation of NF-κB p65 from cytoplasm to nucleus and IκBα degradation induced by Aβ1-42, implying that PGC-1α protects neuroblastoma cells against Aβ-induced neuronal death and neuroinflammation.^447^ From a therapeutic perspective, enhancing PGC-1α levels to boost mitochondrial biogenesis at early stages is a promising pharmacological approach for preventing the onset of AD. However, Dumont et al. illuminated that overexpressing PGC-1α in Tg19959 transgenic mouse exacerbates Aβ and tau accumulation, accompanied by an impairment of proteasome activity.^448^ These paradoxical conclusions reflect that maintaining the delicate balance between PGC-1α expression and its function plays crucial roles in the inhibition of AD and contributes to the design of treatments.

##### PGC-1s in PD

PD, a neurological disorder with evolving layers of complexity, features classical motor dysfunction associated with Lewy bodies (LBs) and dopaminergic neuron loss in the substantia nigra.^449^ Accumulating research illuminated that PGC-1α is involved in the regulation of these deadly physiological processes.

The cardinal motor symptoms of PD correlate with dopaminergic axonal neurodegeneration starting at the striatum, which is then followed by dopaminergic neuronal death in the substantia nigra pars compacta, resulting in dopamine deficiency.^450,451^ Previous studies have illustrated that knockdown of PARIS, a KRAB and zinc finger protein, leads to the mitochondrial respiratory decline and selective loss of dopamine neurons in the substantia nigra. This requires PARIS-induced downregulation of PGC-1α, owing to its ability to directly and endogenously occupy the cis-regulatory elements of PGC-1α.^452,453^ More recently, farnesol has been advocated as a PARIS repressor and it induces the farnesylation of PARIS, further eliminating its DNA binding affinity and preventing its suppression of PGC-1α, thereby antagonizing dopaminergic neuronal loss and behavioral deficits in PD.^454^ The researchers further demonstrated that increased PARIS ubiquitination and proteasomal degradation relieve its repressive effect of PGC-1α, thus alleviating mitochondrial biogenesis.^455^ These indicated that modulating the PARIS-PGC-1α pathway to promote mitochondrial biogenesis and inhibit the loss of dopamine neurons is beneficial in PD. Moreover, a series of studies also confirmed that 1-methyl-4-phenyl-1,2,3,6-tetrahydropyridine (MPTP) induces mitochondrial dysfunction and ROS production, as shown by decreased MMP and ATP levels, as well as increased H_2_O_2_ levels and release of cytochrome c, whereas PGC-1α overexpression partially reverses above phenomenon, thereby alleviating striatal loss of dopamine and progressive impairment of motor coordination. However, PGC-1α deficiency is opposite.^428,430,456–462^ However, there are discrepancies between different studies. Lasting overexpression of PGC-1α contributes to major alterations in the metabolic activity of neuronal cells, which dramatically impairs dopaminergic function, reduces striatal DA content, and enhances susceptibility to MPTP.^429,463^ Sometimes a compensatory loop exists between different molecules due to the artificial manipulation of the key components or regulation of the pathway, which may not precisely imply the real-world conditions. Therefore, while determining the role that PGC-1α plays in dopaminergic neuronal, researchers should also target its mechanism of action in order to lay the foundation for subsequent clinical translational studies.

Another histopathological hallmark of PD is the presence of fibrillar aggregates referred to as LBs containing α-synuclein (α-syn).^464^ PGC-1α null nigral neurons are more prone to degenerate following α-syn overexpression.^465–467^ In contrast, pharmacological activation or genetic overexpression of PGC-1α reduces α-syn oligomerization and α-syn-mediated toxicity.^466^ Additionally, in a zebrafish model of α-syn toxicity, overexpressing of PGC-1α in peripheral sensory neurons inhibits both cell death and axonopathy, thus protecting neurons from α-syn-induced toxicity.^468^ In conclusion, current studies have successfully highlighted the critical role of PGC-1α in the physiology of PD. However, at the molecular level, more exploration is required.

##### PGC-1s in HD

HD is the most frequent autosomal dominant neurodegenerative disorder resulting from an abnormally expanded CAG repeat expansion in the huntingtin (HTT) gene, which confers a predominant toxic gain of function in the mutant HTT protein.^469^ Remarkably, PGC-1α is downregulated in patients with HD and genetic repression of PGC-1α by mutant HTT increases striatal neurodegeneration and motor coordination in mice.^470,471^ Meanwhile, its upstream modulators, including TORCs and downstream transcription factors, such as NRF-1 and TFAM, are also downregulated.^472,473^

At the molecular level, PGC-1α stimulates TFEB, a master regulator of the autophagy-lysosome pathway, thereby promoting HTT protein turnover and elimination.^474,475^ At the organelle level, several publications advocated the role of PGC-1α in HD-related mitochondrial impairment and its potential as a therapeutic target to treat HD.^470,476–478^ PGC-1α upregulation increases mitochondrial mass and rebalances mitochondrial dynamics as well as promoting the mitochondrial fusion.^477^ In BAT from HD mice, a decrement in the numbers of functional mitochondria and ATP/ADP ratio are found. Combined with reduced expression of PGC-1α target genes involved in energy production in BAT, reduced PGC-1α activity possibly leads to a global defect in mitochondrial function in HD.^470^ At the tissue level, PGC-1α plays a role in postnatal myelination by regulating the expression of myelin basic protein (MBP) and cholesterol synthesis in HD. Decreased expression of MBP and deficient myelination are found postnatally in both adult HD models and PGC-1α knockout mice, whereas PGC-1α overexpression increases MBP promoter activity.^479^ These findings raise a possibility that upregulating PGC-1α activity may represent a novel strategy for early therapeutic interventions in HD.

##### PGC-1s in ALS

ALS is a fatal CNS neurodegenerative disease, characterized by the degeneration of both upper and lower motor neurons, which leads to muscle weakness and eventual paralysis.^480^ Notably, in both ALS animal models and ALS patients, the expression of PGC-1α and key mitochondrial genes (e.g. NRF1, NRF2, and TFAM) are downregulated.^481–483^ Liang and colleagues used PGC-1α transgenic mice to cross with SOD1 mutant G93A DL mice and revealed that PGC-1α/G93A DL mice exhibit markedly improved motor activity as compared with G93A DL mice, which is associated with a decreased loss of motor neurons and less degeneration of neuromuscular junctions.^484^ Elevated PGC-1α activity has been validated to sustain mitochondrial biogenesis and muscle function. PGC-1α expression increases mitochondrial energy-producing capacity, thereby making more ATP available for sustained muscle activity.^485^ PGC-1α overexpression dramatically improves motor function and survival, accompanied by reduced blood glucose level and by the protection of motor neuron loss, restoration of mitochondrial electron transport chain activities, and inhibition of stress signaling in the spinal cord.^486^ So far, there are relatively few explorations on the detailed underlying mechanisms regarding PGC-1α in ALS, substantially more studies should be initiated in the future.

#### PGC-1s in kidney diseases

The kidney requires abundant mitochondria to generate energy, thus achieving its inherent and specific tasks, from removing waste from the blood, and reabsorbing nutrients to maintaining fluid and electrolyte balance and regulating blood pressure.^487^ Increasing evidence suggests that dysfunctional renal mitochondria are pathological mediators of different forms of kidney diseases, including acute kidney injury (AKI) and chronic kidney diseases (CKD).^488^ PGC-1s have attracted increased attention in kidney diseases as outstanding regulators situated at the crossroads of mitochondrial energetics. Genetic study has illustrated that PGC-1α directs renal progenitor fate and is necessary for appropriate nephrogenesis in zebrafish.^489^ Of note, PGC-1α is abundantly present in the kidney, but PGC-1β is hardly expressed in the kidney and related research barely exists.^1,16^ In this section, the roles of PGC-1α in kidney diseases are emphasized.

##### PGC-1s in AKI

AKI, formerly termed acute renal failure, is a heterogeneous syndrome featuring by a sudden decrement in the glomerular filtration rate and the rapid loss of the excretory function.^490^ PGC-1α is reported to be downregulated in AKI induced by several factors, including ischemia, sepsis, and toxin.^491–493^

Kidney ischemia-reperfusion injury (IRI), universally occurring in renal transplantation, shock, trauma, and urologic and cardiovascular surgery, is a severe common clinical event leading to rapid kidney dysfunction and AKI.^494^ After 24 h of kidney IRI, the renal PGC-1α expression is downregulated and PGC-1α^-/-^ mice exhibit worsened renal function, increased fat accumulation, and more severe tubular injury. The deeper investigation revealed that PGC-1α promotes NAD de novo synthesis from amino acids by upregulating related enzymes, whereas PGC-1α deficiency weakens the de novo pathway.^491^ In contrast, PGC-1α overexpression or activation following IRI facilitates the recovery of renal function and tubule homeostasis.^495–498^ Closely following this idea, Pan et al. recently found that PGC-1α overexpression enhances the interaction protein between mitochondria and ER and decreases the ER stress regulator hairy and enhancer of split 1, which blocks ER stress and apoptosis, thus protecting renal function during IR-induced AKI.^216^ Besides, FOXO1 inhibits PGC-1α transcription by competing with CREB for binding to transcriptional coactivators CREBBP/EP300. Conversely, FOXO1 inhibition prevents renal tubular epithelial cells apoptosis, ROS overproduction, and IR-induced downregulation of PGC-1α, then improves mitochondrial biogenesis, suggesting that FOXO1 inhibition prevents renal IRI via CREB/PGC-1α-mediated mitochondrial biogenesis.^499^ Brain and muscle ARNT-like 1 (BMAL1), as a pivotal regulator in circadian rhythm, also mediates mitochondrial homeostasis in renal IRI by activating the SIRT1/PGC-1α signal. BMAL1 overexpression significantly restrains apoptosis and oxidative stress, accompanied by the upregulated mRNA and protein levels of SIRT1, PGC-1α, NRF1, and TFAM, whereas SIRT1 inhibitor partially reverses the anti-apoptotic effect of BMAL1 overexpression, reflecting that BMAL1 mediates mitochondrial homeostasis through the SIRT1/PGC-1α axis in kidney IRI.^500^ In addition, some clinical drugs, including N-acetylcysteine, dexmedetomidine, eplerenone, and treprostinil, also exert positive anti-IRI effects on renal tissue by targeting PGC-1α.^501–504^

The kidney is one of the most common organs affected by sepsis and sepsis-associated acute kidney injury (sepsis-AKI) accounts for approximately half of AKI syndrome in ICU, significantly worsening patient prognosis.^505^ By kidney biopsies in patients who died of sepsis-AKI and control patients undergoing tumor nephrectomy, Slikke et al. found that the target genes of PGC-1α, such as TFAM, PINK1, and Parkin, are reduced in sepsis-AKI patients, which likely causes a reduction in mitochondrial mass.^506^ In the sepsis-AKI animal model, downregulated PGC-1α both at mRNA level and protein level are observed.^492,493^ Remarkably, lipopolysaccharide (LPS)-mediated suppression of PGC-1α reduces expression of downstream regulators of mitochondrial biogenesis, electron transport chain proteins, and renal cortical mtDNA content, finally disrupting mitochondrial homeostasis and resulting in renal dysfunction.^493^ Similarly, both in the LPS challenge and cecal ligation and perforation model, PGC-1α expression is proportionally suppressed with the degree of renal impairment. Meanwhile, PGC-1α expression and oxygen consumption decreases when exposed to TNF-α in tubular cells, whereas excessive PGC-1α reverses the latter effect.^199^ Together, these results provide strong evidence that the suppression of PGC-1α is a chief culprit event that affects functional impairment in sepsis-AKI.

In the setting of toxin-mediated AKI, the levels of PGC-1α and its target genes are also downregulated.^507^ In folic acid-induced AKI mice, the inflammatory cytokine TWEAK causes the reduction of PGC-1α expression and loss of MMP. TWEAK promotes histone H3 deacetylation at NF-κB-binding sites at the murine PGC-1α promoter in renal tubular cells and the activation of NF-κB, which impairs mitochondrial function.^507^ The same group further revealed that PGC-1α^-/-^ mice manifest lower survival, more severe renal dysfunction, and an earlier decrement in mitochondrial mass than WT mice. Mechanically, PGC-1α deletion induces higher rates of tubular cell death, compensatory proliferation, expression of proinflammatory cytokines, NF-κB activation, and interstitial inflammatory cell infiltration.^508^ Conversely, in the cisplatin-induced AKI model, overexpression of PGC-1α or PGC-1α activator (ZLN005) treatment blocks cell apoptosis and mitochondrial dysfunction, finally alleviating kidney injury. Furthermore, ZLN005 treatment activates mitophagy, as manifested by increased expression of LC3-II and co-localization between LC3 and mitochondria, and the protective effects are abrogated in TFEB-knockdown cells, suggesting that PGC-1α activation improves mitochondrial dysfunction via TFEB-mediated autophagy.^509^ Additionally, the protective roles of ALDH2, aspirin, and liraglutide via attenuating mitochondrial dysfunction are reliant on PGC-1α-mediated biogenesis.^510–512^

##### PGC-1s in CKD

CKD is characterized by a reduced kidney filtration function, accompanied by nephron loss, inflammation, and extracellular matrix deposition.^513^ With a huge global burden and a prevalence of 10-14%, CKD is now considered a public health priority.^514^ The important roles of PGC-1α in CKD, especially diabetic kidney disease (DKD) and kidney fibrosis, have been noted.

DKD remains one of the fastest-growing causes of CKD and approximately 40% of diabetic patients develop DKD.^515^ Metabolomics analysis indicated that PGC‐1α expressions are downregulated in CKD patients, with a reduction in mitochondrial protein and mtDNA and impaired FAO.^516,517^ In high glucose (HG)-treated rat kidney mesangial cells, FOXO1 inhibition induced by HG downregulates PGC-1α expression, giving rise to mitochondrial dysfunction and ROS generation, while FOXO1 overexpression markedly increases PGC-1α, NRF-1, and Mfn2 expression, and decreases malondialdehyde production and proteinuria.^518^ In line with this, Guo and colleagues illuminated that hyperglycemia leads to the decrement of PGC-1α, which upregulates DRP1 expression, increases mitochondrial fragmentation, and damages network structure, but PGC-1α overexpression counteracts these alterations.^519^ These data suggested that PGC-1α may protect rats against DKD via the attenuation of mitochondrial dysfunction and ROS production. Moreover, the application of mesenchymal stem cells (MSCs) in the treatment of DKD has shown good prospects.^520^ By a coculture system consisting of MSCs and macrophages, it was found that MSCs-derived mitochondria are transferred into macrophages and this transfer stimulates PGC-1α-mediated mitochondrial biogenesis in parallel with the interaction between PGC-1α and TFEB in HG-induced macrophages, leading to the elevated lysosome-autophagy, ultimately ameliorating DKD.^521^ Similar results also exist in the streptozotocin-induced DKD rat model. When MSCs are injected into rats, podocyte injury and PINK1/Parkin-mediated mitophagy are ameliorated, which relies on the activation of the SIRT1-PGC-1α-TFAM pathway.^522^ In addition, some natural products, including resveratrol, berberine, purple rice husk, and formononetin, as well as clinical drugs such as rosiglitazone and rosiglitazone, exhibit protective effects in DKD by performing anti-oxidative effects, anti-apoptosis effects, and preventing mitochondrial dysfunction, in which PGC-1α is a principal hub mediator.^517,523–528^

Kidney fibrosis, characterized by excessive extracellular matrix deposition leading to scarring, is a key determinant of virtually all progressive CKD.^529^ Yang et al. identified PGC-1α as a negative regulator in EMT. Upregulated YY1 expression induced by HG promotes the formation of mTOR-YY1 heterodimer and the nuclear translocation of mTOR-YY1 inactivates PGC-1α by binding to the PGC-1α promoter, which further promotes mitochondrial dysfunction, leading to EMT and tubulointerstitial fibrosis in early DND.^530^ The transcription factor Twist1-induced downregulation of PGC-1α also facilitates kidney fibrosis by reducing FAO and increasing intracellular lipid droplet accumulation, mitochondrial dysfunction, and production of pro-fibrogenic factors.^531^ It is known that inflammation is the initiator and key link to ensuing fibrosis. In the kidney, PGC-1α inhibits the NLRP3 inflammasome to prevent kidney fibrosis. Mechanically, PGC-1α significantly mitigates the oligomerization of NLRP3 with the adapter protein ASC, the release of mtDNA from the mitochondria into the cytosol, and mitochondrial ROS and restores the expression of TNFAIP3 (a negative regulator), thus inhibiting NLRP3 inflammasome complex formation.^209^ In addition, tubule-specific overexpression of PGC-1α ameliorates Notch1-induced kidney injury, as manifested by the restoration of impaired mitochondrial morphology and FAO defect, and the reduction of apoptosis.^87^ The upregulation of PGC-1α by pharmacological approach also alleviates kidney fibrosis via maintaining mitochondrial homeostasis.^532,533^

In aggregate, the functional impacts of PGC‐1α in CKD have been conclusively demonstrated in preclinical studies, as PGC‐1α deficiency shows adverse effects, while genetic PGC‐1α overexpression or pharmacological PGC-1α upregulation is generally beneficial. However, excessive PGC-1α alters mitochondrial properties and induces podocyte proliferation and dedifferentiation, causing collapsing glomerulopathy.^534^ Therefore, controlling the exact levels of PGC-1α and establishing the optimal therapeutic window for PGC‐1α activation is significant to achieve clinical benefits.

#### PGC-1s in motor system diseases

Owing to the high expression of both PGC-1α and PGC-1β in skeletal muscle and the significance of continual supply of ATP in skeletal muscle contraction, it is not unexpected that PGC-1α and PGC-1β have been the research hotspot in skeletal muscle. Recently, the essential roles of PGC-1α and PGC-1β in bone homeostasis have gained considerable popularity and been well-established. For example, PGC-1α mediates osteoblastogenesis and PGC-1β modulates osteoclastogenesis,^36,535–537^ which orchestrates delicate balance between bone resorption and bone formation. Therefore, here we will focus on PGC-1α and PGC-1β in motor system diseases.

##### PGC-1s in osteoarthritis

Osteoarthritis, the most prevalent chronic joint disease, is a major source of pain, disability, and socioeconomic cost worldwide in accordance with the increased aging population and the epidemic of obesity.^538^ Notably, the upregulation of PGC-1α by activating the upstream molecule or coactivating the partners, remarkably reverses impaired mitochondrial biogenesis, oxidative stress, and inflammation in osteoarthritis.^539–544^ Nevertheless, classical drug therapy may be too late to help due to the relatively late diagnosis during the osteoarthritis process. Fortunately, emerging therapies targeting PGC-1α may possess great potential. For instance, mitochondrial transplantation can boost mitochondrial biogenesis in chondrocytes by activating PGC-1α signaling. It was found that the mitochondria of BMSCs could be ingested by rat chondrocytes via intra-articular injection and this mitochondrial transplantation successfully activates PGC-1α signaling, followed by suppressed inflammation, inhibited chondrocytes apoptosis, and improved mitochondrial biogenesis.^545^ More interestingly, zhou et al. conducted a cartilage-targeting dual-drug delivery nano platform (RB@MPMW) composed of rapamycin loaded into the mesopores and bilirubin loaded onto the shell of the metal organic-framework. RB@MPMW can continuously phosphorylate AMPK and further rescue mitochondrial energy metabolism of chondrocytes following IL-1β stimulation via activating the SIRT1-PGC-1α signaling pathway.^546^

##### PGC-1s in DMD

Duchenne muscular dystrophy (DMD), caused by the lack of functional dystrophin protein, is a lethal and progressive disease that leads to difficulties with movement and, eventually premature death.^547^ Amusingly, some gene programs associated with PGC-1α function, including mitochondrial OXPHOS, ROS detoxification, and Ca^2+^ signaling, are dysregulated in DMD,^548–552^ suggesting the feasible connection between PGC-1α and DMD. Importantly, PGC-1α stimulates a powerful program of neuromuscular junctions-linked gene expression both in myotubes and in vivo. Moderately upregulated PGC-1α in skeletal muscle improves fiber damage and fiber necrosis, and decreases serum creatine kinase levels, thereby exerting a beneficial effect in sedentary DMD mice.^553^ When PGC-1α is transferred into already declining muscle, the areas of immune cell infiltration and hypercontracted cells are decreased, and dystrophic muscle is rescued.^554,555^ A recent study indicated that PGC-1α overexpression increases TFEB nuclear localization and lysosome abundance and decreases the severity of DMD in dystrophin-deficient skeletal muscle.^556^

##### PGC-1s in sarcopenia

Sarcopenia, a geriatric disease characterized by a progressive loss of skeletal muscle mass and loss of muscle function, dramatically impinge on life quality and healthcare cost.^557^ Mitochondria usually undergo age-associated changes and their functions are impaired simultaneously, which enables mitochondria dysfunction to be one of the main attributors to sarcopenia progression.^558^ Liu et al. found that the senescence-accelerated mouse prone 8 exhibits typical features of sarcopenia at 40 weeks of age, but the decrement of genes involved in mitochondrial biogenesis (PGC-1α, NRF-1, TFAM, Ndufs8, and Cox5b) and mitochondrial dynamics fission (Mfn2 and Opa1) and autophagic flux are impaired from week 24, suggesting that early alterations of mitochondrial quality control and autophagic flux worsen muscle microenvironment prior to the onset of sarcopenia.^559^ However, PGC-1α overexpression attenuates these age-related increases in mitophagy markers and effectively ameliorates mitochondrial deficits, muscle and adipose tissue functionality, and systemic energy metabolism in aged mice.^560,561^ Genome-wide transcriptional changes analysis from genome-wide transcriptional changes in sarcopenia versus age-matched controls in muscle biopsies revealed that sarcopenia reproducibly manifests low PGC-1α/ERRα signaling, which may explain the global mitochondrial dysfunction including mitochondrial bioenergetic dysfunction, and downregulated OXPHOS and mitochondrial proteostasis.^562^ Notably, Ono et al. established a novel sequential drug screening system and identified an aminoindazole derivative, locamidazole, which can enhance locomotor function, and strengthen muscle and bone by inducing myocyte enhancer factors 2 c (MEF2c) and PGC-1α in a calcium signaling-dependent manner.^537^ Briefly, maintaining an optimal intracellular PGC-1α level and signaling activity contributes to protecting the muscle from sarcopenia.

#### PGC-1s in metabolic disorders

In recent decades, the prevalence and incidence of metabolic disorders, including T2DM, obesity, and metabolic dysfunction-associated steatotic liver disease (MASLD), have dramatically increased worldwide, imposing a staggering burden on whole society as well as individuals.^563^ Some key features of metabolic disorders cover impaired mitochondrial function, a decrement in glucose oxidation and FAO, and insulin resistance.^564^ Courtesy of the principal roles in energy metabolism and insulin sensitivity, PGC-1s may be considered as candidate factors in the etiology and therapeutics of metabolic disorders.

##### PGC-1s in T2DM

There is a growing prevalence of T2DM and its accompanied complications, including DCM, DKD, and diabetic neuropathy in the world.^565^ The pathogenesis is related to a combination of defects in insulin secretion by β-cells and impaired insulin sensitivity in insulin-responsive tissues, such as the liver, skeletal muscle, and adipose tissues.^566^ Over the past two decades, numerous evidence has shown that the expressions of PGC-1α and its downstream responsive genes, which are involved in mitochondrial biogenesis and OXPHOS, are downregulated in human and animal models with T2DM in skeletal muscle and adipose tissue.^567–572^ Conversely, the expression of PGC-1α in the liver are increased in diabetic mice.^573^ Thus, it is not difficult to speculate that the roles of PGC-1s in T2DM depend on the tissue. Importantly, evidence from tissue-specific transgenic or knockout animal models of PGC-1s have supported this notion.

As the principal tissue for the majority of insulin-stimulated whole-body glucose disposal, skeletal muscle is a primary controller of whole-body glucose homeostasis and insulin sensitivity.^574^ As mentioned above, the electro-transfection or overexpression of PGC-1α upregulates GLUT4 expression and glucose uptake in skeletal muscle.^226,227^ Meanwhile, impaired glucose disposal in skeletal muscle leads to insulin resistance and accelerates the development of T2DM.^575^ Notably, PGC-1α hold precise control for glucose disposal by involving in multiple glucose metabolic processes.^228,229^ For example, PGC-1α increases muscle glycogen stores by suppressing glycolytic flux, and downregulating the expression of glycogen phosphorylase and phosphorylase kinase α.^228^ In adipose tissue, reduced expression of PGC-1 and insulin-signaling molecules is associated with adipose tissue dysfunction, which further impairs the systemic insulin response in the insulin-resistant subjects.^570^ These findings emphasize the potential of PGC-1α activation in the treatment of T2DM.

The liver is an important organ in driving gluconeogenesis. In a diabetic model, overexpression of PGC-1α in the liver causes hepatic insulin resistance, manifested by higher glucose production and diminished suppression of gluconeogenesis by insulin.^573^ PCAF is an acetyltransferase of PGC-1α and liver-specific knockdown of PCAF increases PGC-1α activity, which further upregulates blood glucose and hepatic glucose output.^576^ Conversely, selectively inhibiting the gluconeogenic activity of PGC-1α in the liver using SR-18292 (a small molecule) improves hepatic insulin sensitivity and glucose homeostasis in diabetic mice.^577^ Similarly, ZLN005 reduces PGC-1α mRNA levels and gluconeogenesis genes in the liver, while increasing PGC-1α and improving glucose utilization and FAO in skeletal muscle.^19^ In addition, the spexin peptide can repress hepatic gluconeogenesis in both HFD-induced rats and insulin-resistant cells to ameliorate insulin resistance, which also relies on the FOXO1/PGC-1α pathway.^224^ Pancreatic β cells are mainly responsible for synthesizing and secreting insulin. Similar to the liver, overexpressing PGC-1α in isolated rat islets suppresses membrane polarization and induces G6P, thereby inhibiting insulin secretion.^578^ In addition, inducible β-cell PGC-1α overexpression in fetal life leads to decreased β-cell mass, and β-cell hypotrophy, decreased insulin secretion, and damaged glucose tolerance.^579^

Apart from the diabetic complications discussed above, such as DCM and DKD, PGC-1α is involved in the development of other DM-related organ damage, such as diabetic neuropathy and vascular dysfunction. The most prevalent complication is neuropathy and at least 50% of individuals with diabetes develop diabetic neuropathy over time.^580^ Diabetic mice are usually accompanied by peripheral neuropathy, decreased mitochondria and mitochondrial DNA, and increased protein oxidation. Notably, the loss of PGC-1α further aggravates this phenotype and is associated with mitochondria degeneration and increased oxidative stress, while overexpression of PGC-1α in neurons prevents oxidative injury caused by high glucose. These supported the idea that knockout of PGC-1α increases susceptibility to diabetes-induced neuropathy.^581^ In diabetes, the PGC-1α expression in endothelial cells are upregulated. Endothelial PGC-1α effectively inhibits endothelial migration in cell culture and angiogenesis in vivo, leads to aberrant re-endothelialization after carotid injury, blunts wound healing, and reduces blood flow recovery after ischemia. Further mechanism exploration shown that PGC-1α induces Notch signaling, blocks activation of Rac/Akt/eNOS signaling, and renders endothelial cells unresponsive to angiogenic factors, finally contributing to vascular dysfunction in diabetes.^582^ In addition, T2DM disrupts SIRT1/PGC-1α/SIRT3 pathway in the epididymal, which causes a decline of the antioxidant defenses and an increased oxidative damage in that tissue, ultimately leading to impaired male reproductive function.^583^

##### PGC-1s in obesity

Currently, obesity is increasing in an alarming rate (tripling over the past four decades) worldwide,^584^ and causes higher risks of some diseases, including T2DM, MASLD, and cardiovascular diseases.^585^ Continuous expansion of white adipose tissue (WAT) and subsequent ectopic accumulation throughout the body is the chief culprit of obesity, while BAT consumes glucose and triglycerides, thus generating heat.^586^ As described above, PGC-1 was initially cloned from a brown fat cDNA library and shown to drive adaptive thermogenesis in BAT.^1^ In the adipose tissue of obese subjects or mice models, mitochondrial biogenesis regulator PGC-1α, OXPHOS protein levels of complexes I and III, and oxidative metabolic pathways are also reduced.^572,587–589^ Recently, emerging studies have revealed the roles of PGC-1s in adipose tissue.

Obese mice exhibits a marked reduction of PGC-1α, which is accompanied with adipocyte hypertrophy, fibrosis, and decreased mitochondrial respiration.^590^ Kleiner et al. investigated the effects of adipose-specific PGC-1α deficiency on systemic glucose homeostasis. The results showed that when mice with PGC-1α deficiency in WAT are exposed to HFD, they develop insulin resistance and experience decreased suppression of hepatic glucose output.^225^ On the contrary, adipose-specific overexpression of PGC-1α improves mitochondrial biogenesis and respiration, and decreases fasting glucose, blood pressure, and fibrosis. Meanwhile, PGC-1α upregulates the expression of processes associated with the browning of fat tissue, including UCP1, FGF21, and p-AMPK signaling, with a reduction in inflammatory adipokines, NOV/CCN3 expression, and TGFβ. These findings highlight the beneficial impact of adipose-PGC-1α on metabolic disturbances.^590^

As a downstream effector of some transcription factors, PGC-1α mediates their regulatory roles in obesity. For example, Foxj3 overexpression in primary brown adipocytes enhances energy expenditure and improves systemic metabolism on either a chow diet or an HFD. Mechanistically, cold-inducible Foxj3 stimulates the expression of PGC-1α and UCP1, subsequently promoting energy expenditure.^591^ The transcription factor GATA3 mitigates obesity by activating thermogenesis and improving energy expenditure through the upregulation of UCP-1 expression via its interaction with PGC-1α.^592^ TFEB is a basic helix-loop-helix transcription factor. Adipocyte-specific TFEB overexpression protect mice from diet-induced obesity, insulin resistance, and metabolic sequelae. Importantly, adipocyte-specific PGC-1α deficiency also markedly blocks the effects of TFEB overexpression on the induction of browning genes in WAT, as well as diet-induced weight gain and adiposity, suggesting that these metabolic phenotypes of TFEB overexpression are PGC-1α-dependent.^593^ Furthermore, cardiotrophin-like cytokine factor 1 (CLCF1) is a negative regulator of PGC-1α and PGC-1β. Adipocyte-specific CLCF1 transgenic mice develops severe cold intolerance and metabolic dysfunction, partially due to the inhibition of PGC-1α and PGC-1β, which results in impaired mitochondrial biogenesis. This indicates that targeting this pathway restores brown fat activity and systemic metabolic homeostasis in obesity.^594^ Besides, IL-27-IL-27Rα signaling has been found to improve thermogenesis and insulin resistance and protect against obesity. Further investigation showed that IL-27 directly targets adipocytes to elicit the activation of p38 MAPK, thereby enhancing the activation of ATF2 and the expression of PGC-1α and UCP1.^595^

Besides, Kamei et al. found that total energy expenditures increase by up to 1.3 times when the expression of PGC-1β in skeletal muscle is slightly augmented. Consequently, less fat is accumulated and stored.^128^ In 3T3-L1 adipocytes, overexpression of PGC-1β improves insulin sensitivity and mitochondrial function.^176^ In contrast to this, adipose-specific ablation of PGC-1β impairs thermogenesis and reduces the number of contacts between mitochondria and lipid droplets.^596^ These findings demonstrate that PGC-1β contributes to the control of energy balance and provide a potential approach for developing novel anti-obesity drugs.

##### PGC-1s in MAFLD

MAFLD affects up to a third of the global population in parallel with a growing epidemic of obesity and T2DM.^597^ HFD can lead to a state of nonalcoholic fatty liver disease (NAFLD), accompanied by the decreased expression of PGC‑1α and subsequent hepatic inflammation. PGC-1α downregulation promotes phosphorylation of IκBα and subsequent increase in nuclear translocation of p65 NF-κB, ultimately increasing the expression of proinflammatory cytokines.^123^ P2Y2R is a subtype of purinergic P2 receptor. P2Y2R deficiency effectively improves insulin resistance and attenuates hepatic lipid accumulation and injury by enhancing FAO through activation of AMPK signaling and PGC-1α pathway.^598^ In addition, PRMT1, the major protein arginine methyltransferase in mammals, is involved in the transcription, splicing, RNA biology, the DNA damage response, and cell metabolism.^599^ Previous vitro experimental confirmed that PRMT1 promotes hepatic lipogenesis via the TXNIP/PRMT1/PGC-1α pathway.^600^ However, a recent vivo study found that overexpression of PRMT1 in HFD-fed mice alleviates hepatic steatosis by enhancing PGC-1α-mediated FAO via recruitment of HNF4α to the promoter of PGC-1α.^601^ Although the observed results are contradictory, partially due to substantial differences between in vitro and in vivo experiments, all these highlight the important regulatory roles of PGC-1α in MAFLD. Further comprehensive and in-depth exploration will be beneficial in manipulating PGC-1α as a clinical treatment of MAFLD.

Like PGC-1α, PGC-1β plays a dual role in hepatic lipid metabolism. Selective activation of PGC-1β within hepatocytes can prevent liver lipid overload and fibrosis by inducing mitochondrial OXPHOS, FAO and citrate cycle.^239^ The forkhead box protein subfamily member FOXA2 regulates glucolipid metabolism and is closely correlated with hepatic steatosis and NAFLD.^602^ Notably, PGC-1β can coactivate with FOXA2 and modulate hepatic lipid homeostasis. Adenoviral expression of FOXA2 and PGC-1β in the livers of ob/ob mice decreases hepatic triacylglycerols content, increases plasma triacylglycerols concentrations, and promotes apolipoprotein B-containing very-low-density lipoprotein secretion.^603^ However, several studies have suggested that PGC-1β coordinates hepatic lipogenic capacity via interactions with multiple lipogenic transcription factors. Nagai et al. demonstrated that PGC-1β knockdown decreases hepatic de novo lipogenesis, hepatic triglyceride synthesis, and hepatic and peripheral insulin resistance induced by fructose through reducing the expression of sterol regulatory element-binding protein (SREBP)-1 and downstream lipogenic genes in liver.^231^ Furthermore, retinol binding protein 4 (RBP4) induces SREBP-1 activation and consequently accelerates hepatic lipogenesis and plasma triglyceride, but this phenomenon is not observed in PGC-1β knockout mice.^604^ ChREBP is a glucose responsive transcription factor. PGC-1β-mediated coactivation of ChREBP induces genes encoding glycolytic and lipogenic enzymes response to hyperglycemia, whereas liver-specific PGC-1β deficiency impairs the lipogenic response to high glucose conditions.^237^

## Application of Pgc-1s

### Application of PGC-1s in cancer

#### The diagnostic and prognostic value of PGC-1s in cancer in clinical studies

In certain types of cancer, especially those affecting the female reproductive system, alterations in the expression of PGC-1s have manifested significant diagnostic and prognostic value. In OC, the expression of PGC-1α and ERRα exhibits significantly higher in cancer tissues compared to noncancerous tissues, and high expression of PGC-1α is remarkably associated with tumor differentiation. The analysis that combined high PGC-1α and ERRα expression predicts a tendency towards poor cancer-specific survival.^605^ In EC, the expression of PGC-1α and ERRα is higher in highly invasive EC tissues than in less invasive EC and significantly higher than in normal tissues. A single-factor logistic regression analysis confirmed that PGC-1α and ERRα may serve as novel biomarkers for predicting the risk of advanced myometrial invasion.^606^ Similarly, increased levels of PGC-1α in BC patients are correlated with more aggressive cancer characteristics, as well as poorer disease-free survival and overall survival in comparison to patients with lower plasma levels.^607^ Additionally, in CRC, there is a significant correlation between PGC-1α expression and nodal metastasis. The PGC-1α-positive group has reduced overall survival compared to the PGC-1α-negative group, suggesting that PGC-1α represents a biomarker for nodal metastasis and poor prognosis.^279^ In contrast to the above conclusions, high levels of PGC-1α in non-small cell lung cancer are indicative of a positive prognosis. This is supported by the fact that patients with elevated levels of PGC-1α has a median overall survival higher over 24 months, whereas those with low PGC-1α expression only survive for a median of 15.4 months.^608^

#### Pre-clinical studies of PGC-1s in cancer treatment

##### *Natural products or molecules by targeting PGC-1s in cancer*

Currently, no specific drugs targeting PGC-1s in cancer are commercially available in clinics. In pre-clinical studies, the compound that exerts its protective effect by activating PGC-1s or inhibiting PGC-1s are both present.

SR18292, a PGC-1α inhibitor, leads to dysfunction in OXPHOS metabolism, energy exhaustion, and oxidative damage, thus impairing the proliferation and survival of multiple myeloma cells.^609^ Metformin, a first-line drug treatment for T2DM, also increases H_2_O_2_-induced cancer cell death. It downregulates Nrf2 expression by suppressing PGC-1α-mediated PPARγ transcriptional activity, which enhances the susceptibility of WT p53 cancer cells to oxidative stress and therapeutic agents.^610^ Furthermore, the herbal medicine *Paris polyphylla* has been confirmed to inhibit OC. It remarkably decreases the level of PGC-1α, which in turn markedly suppresses the elevated expression of vimentin and recovers the expression of E-cadherin in HG-induced OVCAR-3 cells.^611^ Additionally, isoliquiritigenin, a common herb used in traditional Chinese medicine, inhibits the expression of PGC-1α at protein level and enhances ROS accumulation in gastric cancer cells, but PGC-1α overexpression partly reverses the inhibition of ISL on cell viability.^612^ On the other hand, bouchardatine (an alkaloid derived from *B. Neurococca*) suppresses cancers via PGC-1α activation. It effectively induces a metabolic reprogramming towards aerobic metabolism by upregulating UCP2 through PGC-1α enrichment in its promoter, finally blunting rectal cancer cell proliferation.^613^

##### *Targeting PGC-1s combination with antitumor immunity*

T cell immunotherapy have provided new therapeutic dawn for a wide range of cancer patients, but T cell exhaustion may also represent an inherent impediment in exerting long-lived antitumor effects.^614^ Mitochondria have taken the spotlight as important regulators at different stages of T cell development, while mitochondrial dysfunction is an upstream driver of T cell exhaustion.^615^ Recently, numerous studies have highlighted the potential of targeting PGC-1α in combination with antitumor immunity owing to the predominant roles of PGC-1α in mitochondrial function. PGC-1α activation induced by bezafibrate coactivates NRFs and PPARs, further promoting a series of transcription factors, which enhances FAO and OXPHOS, and mitochondrial expansion, thereby facilitating cytotoxic T lymphocytes (CTL) activation and proliferation.^616^ Then, the same group further found that bezafibrate with PD-1 blockade induces mitochondrial biogenesis and FAO in CD8 + T cells and maintains the number of functional CTLs, which enhances antitumor immunity during PD-1 blockade.^617^ The evidence from another team in lung cancer also supported the similar conclusion.^618^

The enforced expression of PGC-1α promotes CD8 T cell persistence, memory formation, and antigen recall potential, and maintains more robust recall responses to bacterial infection or peptide vaccination. PGC-1α-overexpressing CD8 T cells also has remarkably improved antitumor efficacy.^619,620^ PGC-1α also links epigenetic modification and anti-tumor immunity. Ketogenesis-derived β-hydroxybutyrate, present in CD8+ memory T cells, upregulates Pck1 expression by epigenetically modifying Lys 9 of histone H3 (H3K9) of FOXO1 and PGC-1α, which directs the carbon flow along the gluconeogenic pathway to glycogen and the pentose phosphate pathway, thus promoting CD8 + T-cell memory development.^621^ Besides, Malinee et al. designed a DNA-based epigenetic activator with tri-arginine vector called EnPGC-1, which can stimulate the targeted induction of the PGC-1α/β. Importantly, EnPGC-1 enhances mitochondrial activation, energy metabolism, proliferation of CD8 + T cells, and OXPHOS, thereby improving the longevity and effector functions of killer T cells and augments the efficacy of PD-1 blockade in combination.^622^ Interestingly, an engineered version of PGC-1α containing a point mutation at S571 (PGC-1α^S571A^) has been developed by Lontos and colleagues. PGC-1α^S571A^ transduction endows CAR-T cells potent mitochondrial reprogramming, which drives more effector-like programs and a more long-lived memory state. Therefore, PGC-1α^S571A^ transduced CAR-T cells treatment provides stronger antitumor immunity, and longer survival for all mice.^623^

Taken together, these explorations suggest that targeting PGC-1α combination with antitumor immunity can effectively improve the therapeutic efficacy, success in future clinical trials may benefit cancer patients, especially those who are unresponsive to T cell-based monotherapy.

### Application of PGC-1s in non-cancer diseases

#### The diagnostic and prognostic value of PGC-1s in non-cancer diseases in clinical studies

The altered expression of PGC-1s in various diseases have been described in previous parts. In this section, we focus on examining the connection between PGC-1s gene polymorphism and susceptibility to diseases.

##### Neurological disorders

It has been demonstrated that the coding variant rs3736265 and rs6821591 in PPARGC1A has a significant effect on the age of onset in the population carrying the HD mutation.^624,625^ Moreover, Che et al. discovered the influence of two other single nucleotide polymorphisms (SNP) of PGC-1α in HD. While the minor allele of SNP rs7665116 (g.38570 C), located in the transcribed gene region, is linked to a delay in disease onset, the minor allele of SNP rs2970870 (g.-1437C) in the promoter region contributes to an earlier onset of HD in its homozygous state.^626^ Interestingly, no relation between PGC-1α Gly482Ser polymorphism and oxidative stress biomarker levels is detected in ALS patients under resting conditions. However, during exercise performance, significantly higher lactate levels and greater protein oxidative products are found in AA (Ser482Ser) ALS patients compared to GG (Gly482Gly) and GA (Gly482Ser).^627^

##### Metabolic disorders

The association between PPARGC1A polymorphism and T2DM have been extensively investigated, mainly PPARGC1A Gly482Ser. At first, Kunej et al. found that the AA genotype of the Gly482Ser polymorphism is related to 1.9-times increased risk of T2DM and is considered as a risk factor for the development of T2DM in Caucasians.^628^ The PGC-1α Gly482Ser allele can also predict the conversion from impaired glucose tolerance to T2DM.^629^ Then, over two decades, the researchers conducted a large number of studies. However, conflicting results have also emerged from different studies, which largely depends on population sample sizes, environmental context (area, nation and so on), the tissue-specific functions of the allele, and perhaps even the stage of disease progression.^629–638^

Additionally, in NAFLD, the PPARGC1A rs8192678 risk A allele is associated with an increased risk, even after control for BMI and other confounding factors.^639^ Nevertheless, the Gly482Ser polymorphism of the PGC-1α gene is not associated with the metabolic syndrome in Danish Caucasian subjects.^640^ Interestingly, Huang et al. utilized engineered allele substitution at PPARGC1A rs8192678 to obtain homozygous AA, GG and heterozygous G/A isogenic cell populations. It was shown that the C allele causes reduced levels of PPARGC1A mRNA and PGC-1α protein, along with disrupted dynamics of PGC-1α turnover and activity, which subsequently impacts cellular differentiation and mitochondrial function.^641^ Further studies on the underlying mechanisms in the future may potentially offer novel insights into the discrepancies observed across clinical studies.

#### Pre-clinical studies of PGC-1s in non-cancer diseases treatment

##### Medical treatment

The medical treatments targeting PGC-1s, mainly PGC-1α, have exhibited immense potential in various disease models in preclinical studies. Since a comprehensive presentation of all is too verbose, we will concentrate on a couple of natural products, such as resveratrol,^642–649^ curcumin,^650–654^ berberine,^517,655–660^ quercetin,^661–669^ or clinical drugs, which have been extensively investigated in different pathological models. Other representative compounds, including astragaloside IV,^670–672^ baicalin,^673–676^ dihydromyricetin,^676–681^ isoliquiritigenin,^682,683^ astragalus polysaccharide,^684,685^ dexmedetomidine,^686–689^ will be summarized in Table 1.

**Table 1:** A summary of protective effects of natural or synthesized compounds targeting PGC-1s-related pathway in a variety of diseases

Compounds	Models	Pathway/Targets	Effects	Refs
Resveratrol	Contrast-induced nephropathy	SIRT1/PGC-1α/FOXO1	Reduces oxidative stress, inflammatory cell infiltration, and apoptosis	^642^
	Hyperoxia lung injury	SIRT1/PGC-1α	Upregulates citrate synthase and TFAM expression	^643^
	Diabetic cardiomyopathy	SIRT1/PGC-1α	Ameliorates mitochondrial dysfunction	^644,645^
	Myocardial IRI	SIRT1/SIRT3-Mfn2-Parkin-PGC-1α	Regulates the balance of mitochondria fission-fusion, autophagic flux, and mitochondrial biosynthesis	^646^
	HG-induced kidney injury	SIRT1/PGC-1α	Inhibits oxidative stress and mitochondrial apoptosis pathway and ameliorates mitochondrial function	^647^
	HG-treated retinal	AMPK/SIRT1/PGC-1α	Inhibits ROS-induced apoptosis	^648^
	Hypoxia-treated OC cell	SIRT1/PGC-1α	Recovers SIRT1 and mtDNA expression and antagonizes CoCl_2_-induced VEGF production	^649^
	Neuronal cell injury	PGC-1α	Attenuates autophagy, the release of inflammatory cytokines and ROS generation, and enhances M2 microglial polarization and mitochondrial biogenesis	^690–692^
Curcumin	Liver fibrogenesis	AMPK/PGC-1α	Inhibits collagenα1 and HSCs activation	^650^
	Isoniazid-induced hepatotoxicity	SIRT1/PGC-1α/NRF1	Reduces necrosis, oxidative stress, and inflammation	^651^
	Depression	PGC-1α/FNDC5/BDNF	Promotes neurocyte proliferation and suppresses neuronal apoptosis	^652^
	Tissue repair	PGC-1α/SIRT3/HIF-1α	Inhibits mitochondrial cytochrome c release and apoptosis	^653^
	Cisplatin-induced kidney injury	PGC-1α	Improves mitochondria biogenesis and prevents renal fibrosis and apoptosis	^654^
Berberine	DKD	PGC-1α	Counteracts lipid accumulation, ROS production, mitochondrial dysfunction, and deficient FAO	^517^
	Diabetic nephropathy	C/EBPβ/PGC-1α	Regulates mitochondrial energy metabolism, and inhibits ROS production and apoptosis	^655^
	Diabetic neuropathy	PGC-1α	Attenuates mitochondrial deficits and redox imbalance	^656^
	Fatty liver	PGC-1α	Improves mitochondrial respiratory chain function and insulin signaling	^657^
	Metabolic disorders	AMPK/PGC-1α	Promotes the mitochondrial biogenesis and FAO, and prevents excessive lipid accumulation	^658^
	Aging	AMPK/SIRT1/PGC-1α	Ameliorates aging-related reductions in cognitive ability and muscular function	^659^
	Alzheimer’s disease	GSK3β/PGC-1α	Inhibits tau hyperphosphorylation and neuroinflammation	^660^
Quercetin	Traumatic brain injury	PGC-1α	Inhibits neuronal apoptosis and ameliorates mitochondrial lesions	^661^
	Hypobaric hypoxia-induced memory impairment	SIRT1/PGC-1α	Reduces hippocampus mitochondrial and synaptic lesions	^662^
	H_2_O_2_-induced neuronal damage	SIRT1/PGC-1α	Triggers mitochondrial biogenesis and reduces oxidative stress damage	^663^
	NaIO_3_-induced retinal damage	Nrf2/PGC-1α/SIRT1	Reverses oxidative stress and ROS production	^664^
	Myocardial IRI	SIRT1/PGC-1α	Inhibits cardiomyocyte apoptosis	^665^
	Aluminium-induced oxidative stress	PGC-1α	Inhibits oxidative stress and promotes mitochondrial biogenesis	^666^
	LPS-induced oxidative damage	SIRT1/PGC-1α	Upregulates the mitochondrial membrane potential, and reverse the mitochondrial morphology damage	^667^
	Alcoholic liver disease	PGC-1α	Downregulates redox status, lipid droplets, restores damaged mitochondrial membrane potential, and repairs mtDNA damage	^668^
	Vincristine-induced liver injury	Nrf2/HO-1, NF-kB/STAT3, SIRT1/PGC-1α	Attenuates oxidative stress, apoptosis, and autophagy	^669^
Astragaloside IV	Peritoneal fibrosis	PGC-1α	Enhances mitochondrial synthesis and reduces apoptosis	^670^
	Metabolism disorder	AMPK/PGC-1α	Enhances energy metabolism and inhibits apoptosis	^671^
	Isoproterenol-induced cardiac hypertrophy	NF-κB/PGC-1α	Regulates energy biosynthesis	^672^
Baicalin	Depression	AMPK/PGC-1α	Improves mitophagy level and mitochondrial function	^673,674^
	Insulin resistance	p38 MAPK/PGC-1α	Decreases body weight, HOMA-IR, and alleviates HFD-induced glucose intolerance, hyperglycemia, and insulin resistance	^675^
	Pulmonary hypertension	PGC-1α	Ameliorates angiogenesis	^676^
Dihydromyricetin	Diet-induced obesity	IRF4/PGC-1α	Reduces body weight, decreases WAT mass, improves glucose and lipid metabolic disorders, and ameliorates hepatic steatosis	^677^
	Gentamicin-induced ototoxicity	PGC-1α/SIRT3	Protects cells from apoptotic death by inhibiting ROS accumulation	^678^
	Dexamethasone-induced muscle atrophy	PGC-1α	Stimulates mitochondrial biogenesis and promotes mitochondrial fusion, rescues the reduced mtDNA content, improves mitochondrial morphology	^679^
	Type 2 diabetes	AMPK/PGC-1α/SIRT3	Activates insulin signaling and increases glucose uptake in skeletal muscle	^680^
	Alcoholic liver disease	AMPK/SIRT1/PGC-1α	Increases TFAM expression, hepatic ATP concentrations, and induces mitochondrial expression of respiratory complex III and V	^681^
Isoliquiritigenin	LPS/D-GalN-induced acute liver failure	PGC-1α/Nrf2	Improves the ability of anti-oxidative stress, alleviates inflammatory reaction and apoptosis	^205^
	Alcoholic liver injury	miR-23a-3p/PGC-1α	Promotes fatty acid metabolism and inhibits the ROS	^682^
	Nonalcoholic fatty liver disease	miR-138-5p/PGC-1α	Promotes lipid metabolism and inhibits inflammatory response	^683^
Astragalus polysaccharide	Cardiac hypertrophy	TNF-α/PGC-1α	Improves the cardiac hemodynamics	^684^
	Insulin resistance	SIRT1/PGC-1α/PPARα	Suppresses abnormal glycolipid metabolism and insulin resistance	^685^
Dexmedetomidine	Acute kidney injury	PGC-1α/STAT1/IRF-1	Inhibits mitochondrial damage and inflammation	^502^
	Traumatic brain injury	PGC-1α	Relieves encephala edema and neuron cell apoptosis and increases behavioral function	^686^
	Intracerebral hemorrhage	PGC-1α	Increases GPX and SOD levels and reduces MDA and nitric oxide levels	^687^
	Doxorubicin-cardiotoxicity	PGC-1α	Attenuates mitochondrial dysfunction, oxidative stress, and apoptosis	^688^
	OGD/R	PPARδ-AMPK-PGC-1α	Enhances the cell viability and decreases ROS production	^689^
Melatonin	OGD/R; Myocardial IRI	PGC‑1α/Nrf2; AMPK/PGC1α	Represses oxidative stress and inflammation	^694,695^
	Cardiac hypertrophy	PGC-1α/MICU1	Ameliorates ROS generation and promotes mitochondrial function	^696^
	Ischemia	PGC-1α	Promotes OXPHOS and angiogenic ability of MSCs	^697^
	Kidney injury	AMPK/SIRT1/PGC-1α	Relieves oxidative stress, mitochondrial dysfunction, and apoptosis	^698^
	Diabetic myocardial IRI	PGC-1α	Improves mitochondrial quality control, alleviates diabetic cardiomyopathy, and reduces myocardial vulnerability to IRI	^166,699,700^
	Chromium-induced lung injury	SIRT1/PGC-1α/Nrf2	Reduces oxidative stress and inflammatory mediators and inhibits cell apoptosis	^701^
	Rotenone-induced mitochondrial deficiency	SIRT1/PGC-1α	Abrogates mitochondrial dysfunction, ATP deficiency, oxidative stress, and apoptosis	^702^
	Cadmium-induced kidney injury	SIRT1/PGC-1α	Attenuates Drp1- and Fis1-mediated mitochondrial fission and mitochondrial oxidative stress	^703^
	Bisphenol A-induced colon injury	SIRT1/PGC-1α	Restores the mitochondrial dynamic balance and activates the Nrf2 antioxidant axis	^704^
Metformin	High-glucose environment	AMPK/SIRT1/PGC-1α	Promotes cell proliferation, enhance GSIS, and suppresses apoptosis	^707^
	p53 cancer cells	SIRT1/ PGC-1α/Nrf2	Increases the susceptibility of p53 cancer cells to oxidative stress and TRAIL-induced apoptosis	^610^

##### Resveratrol

Both preclinical experiments and clinical trials of resveratrol achieved tremendous benefits in a variety of human diseases, such as diabetes, cardiovascular diseases, neurodegeneration, and cancers, in which PGC-1α is a potential target.^642–645^ In terms of cardiovascular disease, resveratrol reestablishes the balance of mitochondria fission-fusion and regulates autophagic flux and mitochondrial biosynthesis through the SIRT1/SIRT3-Mfn2-Parkin-PGC-1α pathway in myocardial IRI.^646^ Asymmetric dimethylarginine and HFD promotes PGC-1α acetylation and results in DM, whereases resveratrol treatment remarkably reverses altered PGC-1α expression and acetylation in the myocardium, thus ameliorating cardiac and mitochondrial dysfunction.^644,645^ In CNS, resveratrol exerts neuroprotective effects against neuronal cell injury via attenuating autophagy, suppressing the release of inflammatory cytokines and ROS generation, and enhancing M2 microglial polarization and mitochondrial biogenesis.^436,690–692^ Under HG induced-kidney, SIRT1 and PGC-1α are downregulated, which exacerbates oxidative stress, activates mitochondrial apoptosis pathway, and impairs mitochondrial function, while resveratrol can partially offset these phenomena through the SIRT1/PGC-1a axis.^647^ In addition, resveratrol can also trigger the AMPK/ SIRT1/PGC-1α pathway to inhibit ROS-induced apoptosis in HG-treated retinal capillary endothelial cells.^648^ During hyperoxia, the activation of the SIRT1/PGC-1α signaling pathway by resveratrol attenuates lung injury and VEGF induction.^649^ Briefly, resveratrol, as a classical agonist of SIRT1, combats oxidative stress, inflammation, apoptosis, and mitochondrial dysfunction by activating the SIRT1/PGC-1α pathway, eventually providing protection against various diseases.

##### Curcumin

Curcumin, a crucial polyphenol present in Curcuma longa L. rhizome, exemplifies a promising traditional medicinal agent. Recent studies have revealed anti-apoptotic, anti-oxidative, and antidepressant properties of curcumin that arise from its modulation of PGC-1α. In the liver, curcumin activates AMPK and increases PGC-1α expression, then inhibiting collagenα1 and hepatic stellate cells (HSCs) activation, thus effectively preventing liver fibrogenesis.^650^ Severe hepatotoxicity greatly limits the application of isoniazid, a first-line drug in tuberculosis. Li et al. found that curcumin alleviates isoniazid-induced hepatotoxicity by upregulating the SIRT1/PGC-1α/NRF1 pathway.^651^ In chronic unpredictable mild stress-induced depression-like behavior, curcumin supplementation promotes neurocyte proliferation and inhibits neuronal apoptosis, while PGC-1α inhibitor SR18292 reverses the beneficial effects of curcumin on depressed rats.^652^ Of note, curcumin combined with other treatment methods shows tremendous treatment effects. For instance, curcumin combined with hypoxic preconditioning obviously promotes cell survival, improves mitochondrial function in BMSCs, and inhibits mitochondrial cytochrome c release as well as consequent apoptosis signal. However, PGC-1α RNAi simulates mitochondrial superoxide and H_2_O_2_ production in hypoxia.^653^ Co-treatment of curcumin with cisplatin promotes apoptosis and activates endothelin-1 clearance in the SKOV3 cell (Human OC cell line) and OC rat model, thus preventing renal fibrosis. These shed light on curcumin as a therapeutic adjuvant in the clinical setting.^654^

##### Berberine

Berberine is a representative isoquinoline alkaloid as well as an eminent component of traditional Chinese medicine for more than 2000 years.^693^ Berberine has the ability to suppress many diabetic complications.^517,655,656^ In db/db mice, berberine treatment inhibits lipid disorder-induced podocyte damage and development of DKD by counteracting lipid accumulation, ROS production, mitochondrial dysfunction, and deficient FAO, in which PGC-1α-mediated mitochondrial bioenergetics perform a key role.^517^ In neuronal cells, berberine treatment facilitates PGC-1α-mediated mitochondrial biogenesis and redox imbalance, thereby inhibiting diabetic neuropathy.^656^ Moreover, berberine affects the lipid deposition of skeletal muscle and liver.^657,658^ Mechanically, berberine activates the AMPK/PGC-1α pathway, thus promoting mitochondrial biogenesis and improving FAO, eventually preventing excessive lipid accumulation.^658^ Berberine also ameliorates aging-related reductions in cognitive ability and muscular function, which benefits from the activation of the AMPK/SIRT1/PGC-1α pathway.^659^ Meanwhile, it represses tau hyperphosphorylation and neuroinflammation, which is attributed to the regulation of the GSK3β/PGC-1α signaling pathway in APP/PS1 mice.^660^

##### Quercetin

Pre-clinical experiments of quercetin revealed their therapeutic efficacy in T2DM, AD, liver injury, and cardiac diseases. In neuronal cells, quercetin remarkably inhibits neuronal apoptosis and ROS generation, reestablishes mitochondrial biogenesis and dynamics, and ameliorates mitochondrial function by activating PGC-1α-related pathway.^661–663^ In the ARPE19 cells, NaIO_3_ exposure changes the retinal structure and suppresses pupil constriction, while quercetin treatment inhibits the generation of mitochondrial ROS, which is dependent on increased levels of deacetyl-SOD2 through the Nrf2-PGC-1α-SIRT1 signaling pathway.^664^ During myocardial IRI, quercetin can also mitigate apoptosis via SIRT1/PGC-1α signaling.^665^ In other injury models induced by LPS, aluminium, ethanol, or vincristine, quercetin treatment alleviates oxidative stress, apoptosis, autophagy, and mitochondrial homeostasis, accompanied by increased levels of PGC-1α.^666–669^ In summary, quercetin possesses powerful organ protective functions by targeting PGC-1α and may represent a therapeutic strategy.

##### Melatonin

Melatonin, the primary circadian output signal from the brain, is uncommonly effective in anti-oxidative stress, anti-inflammatory, anti-apoptosis, and anti-fibrosis, thus offering protection against a wide variety of diseases.^694–698^ For instance, in the OGD/R or myocardial IRI model, melatonin plays protective roles via the inhibition of oxidative stress and inflammation by regulating the PGC‑1α/Nrf2 and PGC‑1α/TNF‑α signaling pathways.^694,695^ Furthermore, in the setting of diabetic myocardial IRI, melatonin effectively improves mitochondrial quality control, alleviates diabetic cardiomyopathy, and hence reduces myocardial vulnerability to IRI through the SIRT1-PGC-1α or AMPK-PGC-1α pathway.^166,699,700^ The beneficial roles of melatonin in various toxin-caused organ injuries, such as chromium-induced lung injury, di-phthalate-induced granulosa cells apoptosis, CCl_4_-induced liver fibrosis, and rotenone-induced early porcine embryos, have been sufficiently demonstrated, which relies on the activation and increased expression of PGC-1α.^701–704^

##### Metformin

Metformin is currently the first-line and wide-spectrum drug treatment for T2DM and its inducible effect of AMPK is adequately documented. Thus, it is well established that metformin upregulates PGC-1α via AMPK phosphorylation under different experimental models.^52^ In the context of ischemic diseases occurring in the brain and heart, metformin pretreatment modulates mitochondrial energy metabolism and apoptotic cell death pathways through AMPK activation.^705,706^ When exposed to a high-glucose environment, metformin can promote INS-1 cell proliferation, enhance glucose-stimulated insulin secretion (GSIS), and suppress apoptosis by activating AMPK/SIRT1/PGC-1α signal pathway, up-regulating irisin expression, and inducing autophagy.^707^ Besides, metformin protects against gluco- and lipotoxicity-induced osteoblast apoptosis and reverses T2DM-associated deterioration in skeletal health, whereas depletion of PGC-1α abolishes this protective effect.^708^

##### Exercise training treatment

PGC-1α was acknowledged as a transcriptional coactivator induced by exercise as early as it was discovered.^709–713^ Terada et al. further illuminated that exercise stimulates PGC-1α expression at least via two distinct mechanisms, including AMPK activation and Ca^2+^ elevation.^711^ Moreover, the increased protein abundance in LKB1 and PGC-1α with endurance and interval training is responsible for maintaining the training-induced increases in mitochondrial mass.^712^ Exercise training has been confirmed to play important roles in muscle function, insulin sensitivity, mitochondrial biogenesis, angiogenesis, and unfolded protein response by regulating PGC-1α.^214,714,715^ Strikingly, PGC-1β declines rather than increases in prolonged exercise, which is more obvious when glycogen is not resynthesized to rest levels,^716^ in which the underlying mechanisms and causes are thought-provoking and need additional work to address. As Neto et al., published a wonderful review regarding the multifaceted and multi-systemic actions of physical exercise on PGC-1α signaling in just past 2023 April,^717^ we do not summarize the related frontier-of-knowledge data again herein.

##### Caloric restriction treatment

CR is a powerful and noninvasive intervention method to extend both life- and health span.^718^ PGC-1α, as a center of energy metabolism and mitochondrial OXPHOS, represents one of the most significant molecules that links the benefits of CR to the improvement of healthy conditions by limiting ROS generation, regulating insulin resistance, and mitochondrial function. The first and foremost investigation regarding the effects of CR in PGC-1α revealed that the levels of mtDNA, PGC-1α, NRF-1, and TFAM are upregulated in CR mice compared with ad libitum mice in adipose tissue, brain, heart, and liver.^719^ Soon afterward, Baker et al. reported that CR attenuates the decrement of PGC-1α gene expression with aging.^720^ Specifically, the potential mechanisms may involve that the suppression of GSK3β induced by CR to protect PGC-1α from intranuclear proteasomal degradation and the induction of SIRT1 by CR to enhance the transcriptional activity of PGC-1α.^66^ A subsequent series of research validated that CR upregulates the expression of PGC-1α as well as its target genes in mice, thereby supporting optimal energy metabolism and biochemical adaptation and performing protective roles in distinct diseases.^721–725^ However, another study found that CR downregulates the expression of the PPAR superfamily both in the muscle of normal and long-lived growth hormone receptor/binding protein knockout mice.^726^ In addition, the levels of PGC-1α, NRF-2, and ROS exhibit no alterations in rat liver of 40% restriction of dietary amino acids.^727^ More interestingly, short-term CR upregulates the mRNA levels of GLUT4, PGC-1α, and SIRT3 in cardiac muscles in young but not old rats, and downregulates only PGC-1α expression in skeletal muscles.^728^ Therefore, these conflictive results might be attributed to tissue type-dependent effects and age context-dependent influence of CR on PGC-1α. Moreover, the specific implementation plan, including varied caloric intake, variable feeding frequency, diet composition, and detection time point might also be partly responsible for the inconsistent phenomena.

Notably, although CR does not increase mitochondrial content, the adaptive induction of PGC-1α by CR maintains a functionally ‘efficient’ electron transport system and mitochondria in skeletal muscle, reflecting the importance of PGC-1α for the ability of dietary restriction to counteract the age-related decrement in mitochondrial respiration.^723^ Nevertheless, a normal improvement in glucose homeostasis in response to CR is observed in mice lacking skeletal muscle PGC-1α. Together with the results that muscle-specific overexpression of PGC-1α does not enhance metabolic improvements in response to CR, it is thought that skeletal muscle PGC-1α is not necessary for the whole-body benefits of CR.^729,730^ Obviously, consensus regarding the metabolic benefits of upregulated PGC-1α levels remains to be established. In other words, the reciprocity between PGC-1α levels, mitochondrial performance, and metabolic homeostasis may be more complex than previously, and more attention should be paid to decipher sophisticated interplay.

## Conclusion remarks and future directions

**Fig. 8:**
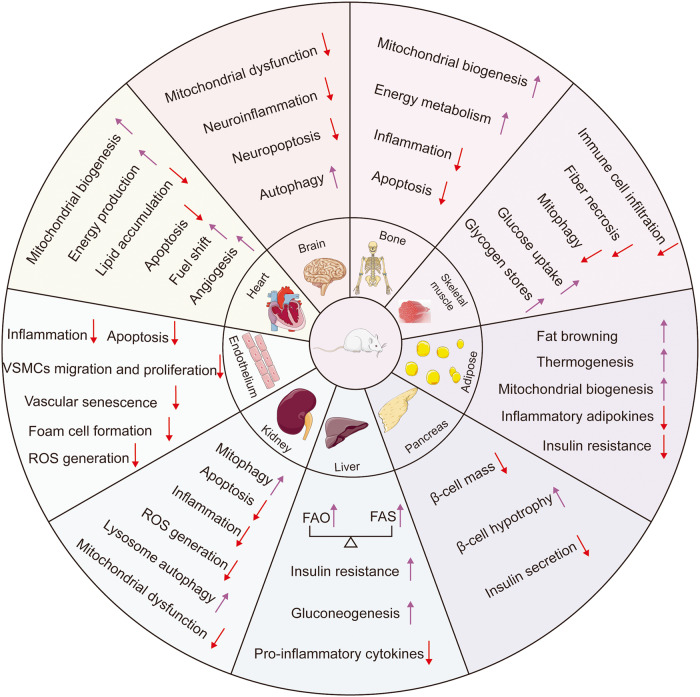
The important regulatory roles of PGC-1α overexpression or activation in various organs. PGC-1α play important regulatory toles in various cellular events, including inflammation, apoptosis, mitochondrial function, and ROS generation, as well as some metabolic processes, including gluconeogenesis and glycogen stores in different organs or tissues, thus widely involving in the occurrence and progression of many diseases

Taken together, substantial insights into the PGC-1s family have illustrated their important functions and regulatory roles in the development of various diseases in the past few decades (Table 2, Fig. 8). Here, this review presents a complex regulatory network of the PGC-1s upstream, parallel, and downstream as well as the presently essential functions of PGC-1s, establishes an overview regarding the effects of PGC-1s in health and diseases, and introduces known therapeutic strategies targeting PGC-1s in pre-clinical experiments, which may thereby contribute to increasing our understanding of PGC-1s and tap the possible application of PGC-1s as novel therapeutic targets. Despite the encouraging progress in this area, some other directions in basic research and clinical applications of PGC-1s are worthy of attention.

**Table 2:** A summary of the functions of PGC-1s in different organs and diseases models

Disease models	Intervention	Main effects	Refs
Colorectal cancer	Intestinal-specific PGC-1β transfection	A peculiar intestinal morphology with very long villi and greater tumor susceptibility	^292^
Heart failure	Heart-specific PGC-1α knockout	Impairs mitochondrial respiration, energy metabolism, and Ca^2+^-handling and profound cardiac dysfunction	^361–363^
Heart failure	Heart-specific PGC-1α transfection	Increases mitochondrial damage and ROS insult	^366^
Peripartum cardiomyopathy	Heart-specific PGC-1α knockout	Enlarges left ventricular end-diastolic and end-systolic dimensions, and depresses cardiac contractile function	^390^
Parkinson’s disease	Dopaminergic neurons-specific transfection of PGC-1α	Elevates mitochondrial antioxidants and reduces loss of dopamine	^456^
Parkinson’s disease	Dopaminergic neurons-specific knockdown of PGC-1α	Leads to mitochondrial dysfunction	^459^
Parkinson’s disease	Microglial cells-specific knockdown of PGC-1α	Inhibits microglia activity, and reduces both M1 and M2 microglial activities.	^462^
Kidney fibrosis	Tubule-specific overexpression of PGC-1α	Alleviates mitochondrial morphology and FAO defect, and reduces apoptosis	^87^
Type 2 diabetes mellitus	Skeletal muscle-specific overexpression of PGC-1α	Upregulates expression of GLUT4 and increases glucose uptake in skeletal muscle-	^226,227^
Type 2 diabetes mellitus	β-cell-specific overexpression of PGC-1α	Decreases β-cell mass, and β-cell hypotrophy, decreases insulin secretion, and impairs glucose tolerance	^579^
Obesity	Adipose-specific PGC-1α knockout	Leads to insulin resistance and decreases the suppression of hepatic glucose output	^225^
Obesity	Adipose-specific overexpression of PGC-1α	Improves mitochondrial biogenesis and respiration, decreases fasting glucose, blood pressure, and fibrosis.	^590^

Although the theme of this review is the PGC-1s family, PRC receives litter attention as the related research is few very much. Meanwhile, PGC-1β is also less relatively characterized compared to PGC-1α. Based on the current research, PGC-1α and PGC-1β have overlapping and distinct features and functions with each other. First, they manifest a similar expression pattern, as shown by extensively elevated expression in tissues demanding high energy requirements, such as the heart, skeletal muscle, and BAT. However, PGC-1α is highly inducible by different physiological or pharmacological cues, while PGC-1β seems to be less responsive to such stimuli. Second, PGC-1α can be regulated by several transcription and post-translational modifications, but the related report about PGC-1β is less. As sequence conservation among different members of the family, it can be inferred that many modulation modes of PGC-1α are also valid for PGC-1β and PRC. Last but not least, the functions between PGC-1α and PGC-1β are not always redundant. To be specific, both PGC-1α and PGC-1β significantly affect mitochondrial oxidative metabolism. Nevertheless, their functional heterogeneity is particularly evident in the liver. PGC-1α principally controls the gluconeogenesis genes, such as PEPCK and G6P, in response to fasting or feeding. Contrary to PGC-1α, PGC-1β predominately regulates hepatic lipid metabolism by interacting with ChREBP and SREBP.^237,731^ Therefore, a more complete understanding among different members of the PGC-1s family will be helpful for the development of innovative treatment.

As discussed above, except for the accepted double-edged sword of PGC-1s in cancer, upregulated PGC-1s expressions in other pathological processes are also not advantageous. For example, in the heart, sustaining physiological levels of PGC-1α expression following POH does not prevent mitochondrial and contractile dysfunction.^376^ However, even though the overexpression of PGC-1α is at a moderate level, enhanced mitochondrial biogenesis leads to significantly greater acute mortality in pressure-overloaded mouse hearts.^366^ Recently, Zhu et al. revealed that PGC-1α overexpression exacerbates cardiac degeneration and shortens lifespan in WT mice, but a favorable longevity-extending effect is observed in a third generation of telomerase-deficient mouse model.^367^ A similar conclusion also exists in the effects of PGC-1α on insulin resistance. Although it is widely recognized that PGC-1α is an important partner in combating insulin resistance,^732^ muscle-specific PGC-1α overexpression mice are more likely to develop insulin resistance, which comes from decreased insulin-stimulated muscle glucose uptake.^733^ Therefore, the following questions remain to be addressed in future investigations 1) probing the extent to which PGC-1α takes part in modulating energy homeostasis under physiological conditions, 2) exploring the mechanisms that PGC-1α activity alters in a diverse array of diseases, 3) determining the appropriate levels of PGC-1α to achieve health benefits under different pathologic condition, and 4) developing the methods to precisely tuning the expression of PGC-1α.

As for the clinical application of pharmacological methods targeting PGC-1α, some natural products like berberine, resveratrol, and curcumin, have shown protective effects in preclinical studies. However, they are still in a very embryonic state. Not only clinical trials but also multiple limitations of natural products such as low bioavailability, inadequate biological stability, and poor aqueous solubility, are needed to be further addressed. Additionally, these natural products have been widely reported to act on other targets, such as PI3K, AMPK, Nrf2, NF-κB, etc.^734–736^ Therefore, the observed therapeutic effects of these drugs might be unintended consequences rather than specific targeting of PGC-1s. The clinical drugs that have been approved, such as metformin and melatonin are promising candidates. The hurdle of expanding their clinical indications by targeting PGC-1α involves toxicology analyses, dosing, and formulation optimization. In addition, PGC-1α activator ZLN005 and inhibitor SR-18292 have been developed and applied in animal experiments,^19,509,577,737–739^ However, gaps and differences exist between rodent models and humans, thus more clinical trials are required.

Moreover, the pleiotropic effects of PGC-1α also depend on the tissue type. Specifically, the whole-body overexpression of human PGC-1α increases the expression of HNF4α and gluconeogenic enzymes PEPCK and G6P in the liver, and causes hepatic insulin resistance, while insulin sensitivity is improved in muscle.^573^ Likewise, short-term CR and endurance training differently affect energy metabolism and mitochondrial biogenesis in the cardiac and skeletal muscle.^370,740^ In one aspect, this emphasizes the necessity of conducting tissue-specific deficiency or overexpression models. In another aspect, from a therapeutic view, achieving targeted delivery to tissues or organs without affecting others contributes to avoiding unsatisfactory side effects. For example, Hao et al. designed 4,6-diamino-2-pyrimidinethiol-modified gold nanoparticles (D-Au NPs) and investigated its effect on intestinal mitochondria and studied the regulatory role of D-Au NPs on mitochondria metabolism-related disease. They found that D-Au NPs enhances the intestinal mechanical barrier by improving the antioxidation capability of mitochondria, and maintaining intestinal cellular homeostasis via the activation of AMPK and PGC-1α, as well as with its downstream signaling (UCP2 and DRP1).^741^ As described above, the cartilage-targeting dual-drug delivery nano platform (RB@MPMW) can achieve the sequential release of two agents (rapamycin and bilirubin) via near-infrared (NIR) laser irritation, thereby rescuing mitochondrial energy metabolism of chondrocytes via activating SIRT1-PGC-1α signaling pathway.^546^ More importantly, a nanoparticle that carries endothelial-specific PGC-1α expression plasmid was developed. Endothelial-specific overexpression of PGC-1α remarkably impedes endothelial to mesenchymal transition of pulmonary arterial endothelial cells and reduces vascular muscularization, thereby attenuating the development of pulmonary hypertension.^742^ With the development of drug screening technologies and targeted drug delivery systems, further investigations will facilitate improved applications of PGC-1α in clinical treatment.

With respect to the non-pharmacological methods mentioned above, making a personalized therapy plan based on a specific analysis and diagnosis of each individual is of vital importance. Of note, combined therapy is an emerging therapy and successfully alleviates the developments in animal models and clinical trials. For example, melatonin supplement integrated with exercise preserves mitochondrial function and represses oxidative stress, thus preventing cardiac injury.^743^ Besides, both CR in combination with high-intensity interval training and high-intensity interval training alone upregulates the levels of PPARγ and PGC-1α in visceral adipose tissue of obese rats, thus boosting the browning of visceral fat and ultimately weakening fat, while the former is more effective.^744^ Undeniably, a plausible strategy that combines moderate CR, physical activity, and pharmacological intervention represents one of the best ways to prevent diseases.

Interestingly, at 3 days post fertilization in zebrafish model, PGC-1α and PGC-1β knockdown decrease the transcript levels of citrate synthase, 3-hydroxyacyl-CoA dehydrogenase, and medium-chain acyl-coenzyme A dehydrogenase.^745^ Additionally, Kurchaba et al. discovered that the disruption of PGC-1α gene expression in striated muscle results in 4~fold increased mRNA levels of PGC-1α in mixed skeletal muscle and an opposite 4~fold downregulation in cardiac muscle. Meanwhile, two mitochondrial lipid transporters, CPT-1 and CPT-2, are strongly induced in mixed skeletal muscle and several transcriptional regulators (ERRα, NRF-1, and PGC-1β) are decreased without altering metabolic gene expression.^746^ This suggest that a mutation of PGC-1α promoter increases resting metabolism, translating into an enhanced mitochondrial oxidative capacity and FAO in adult zebrafish muscle.^746^ Therefore, zebrafish may serve as unique biomedical models for the investigation about the roles of PGC-1s in metabolic disorders.

In summary, the PGC-1s family is a promising target for the prevention and management of diseases. As big gaps of knowledge about the PGC-1s family still exist, especially about PGC-1β and PRC, more extensive research and the deeper elaborate mechanisms of other underlying roles for PGC-1s in the cellular events and pathological processes are hopefully warranted in the future.
